# Prospective Study of Serum Uric Acid Levels and First Stroke Events in Chinese Adults With Hypertension

**DOI:** 10.3389/fphys.2021.807420

**Published:** 2021-12-23

**Authors:** Feng Hu, Longlong Hu, Rihua Yu, Fengyu Han, Wei Zhou, Tao Wang, Linjuan Zhu, Xiao Huang, Huihui Bao, Xiaoshu Cheng

**Affiliations:** ^1^Department of Cardiovascular Medicine, The Second Affiliated Hospital of Nanchang University, Nanchang, China; ^2^Jiangxi Provincial Cardiovascular Disease Clinical Medical Research Center, Nanchang, China; ^3^Department of General Practice Medicine, Xucun Town Health Center, Wuyuan, China; ^4^Center for Prevention and Treatment of Cardiovascular Diseases, The Second Affiliated Hospital of Nanchang University, Nanchang, China

**Keywords:** serum uric acid, first stroke, Chinese adults, hypertension, aging

## Abstract

**Objectives:** We investigated the association between serum uric acid (SUA) levels and the risk of the first stroke in Chinese adults with hypertension.

**Methods:** A total of 11, 841 hypertensive patients were selected from the Chinese Hypertension Registry for analysis. The relationship between SUA levels and first stroke was determined using multivariable Cox proportional hazards regression, smoothing curve fitting, and Kaplan–Meier survival curve analysis.

**Results:** During a median follow-up of 614 days, 99 cases of the first stroke were occurred. Cox proportional hazards models indicated that SUA levels were not significantly associated with the first stroke event [adjusted-hazard ratio (HR) per SD increase: 0.98, 95% CI 0.76–1.26, *P* = 0.889]. In comparison to the group without hyperuricemia (HUA), there were no significantly higher risks of first stroke events (adjusted*-*HR: 1.22, 95% CI 0.79–1.90, *P* = 0.373) in the population with HUA. However, in the population less than 60 years old, subjects with HUA had a significantly higher risk of the first stroke than the population without HUA (adjusted-HR: 4.89, 95% CI 1.36–17.63, *P* = 0.015). In subjects older than 60 years, we did not find a significant relationship between HUA and first stroke (adjusted-HR: 0.97, 95% CI 0.60–1.56, *P* = 0.886). Survival analysis further confirmed this discrepancy (log-rank *P* = 0.013 or 0.899 for non-aging or aging group).

**Conclusion:** No significant evidence in the present study indicated that increased SUA levels were associated with the risk of first stroke in the Chinese adults with hypertension. Age played an interactive role in the relationship between HUA and the first stroke event.

## Introduction

Serum uric acid (SUA) is a final enzymatic product of purine metabolism, and several gradual changes have led to the higher SUA levels in humans than in other mammals. This may hint at an evolutionary advantage owing to its antioxidant properties by acting as a scavenger of hydroxyl radicals and chelation of transition metals (Sautin and Johnson, 2008; Sinha et al., 2009). During the past three decades, the role of hyperuricemia (HUA) on stroke incidence across populations has been controversial (Maloberti et al., 2020). Most prospective studies have assessed the association of HUA with stroke incidence in the general population (Chien et al., 2005; Bos et al., 2006; Gerber et al., 2006; Hozawa et al., 2006; Tu et al., 2019; Li J. et al., 2020), patients with hypertension (Shi et al., 2017; Zhang et al., 2020), atrial fibrillation (Chao et al., 2014), and non-insulin-dependent diabetes mellitus (DM) (Lehto et al., 1998). Most reported a positive association between HUA and stroke risk (Lehto et al., 1998; Chien et al., 2005; Bos et al., 2006; Chao et al., 2014; Tu et al., 2019; Li J. et al., 2020; Zhang et al., 2020), some reported an insignificant relationship (Gerber et al., 2006; Hozawa et al., 2006; Shi et al., 2017). Two meta-analytical reviews of prospective observational studies suggest that HUA may modestly increase the risks of both stroke incidence and mortality (Kim et al., 2009; Li et al., 2014).

Furthermore, some previous studies indicated that the influence of SUA on stroke was due to the secondary association of SUA with other established etiological risk factors, such as hypertension, arterial stiffness, obesity, and hyperinsulinemia (Lehto et al., 1998; Hozawa et al., 2006; Ishizaka et al., 2007; Liang et al., 2009; Shi et al., 2017; Chaudhary et al., 2020). Multiple lines of evidence from epidemiological (Perlstein et al., 2006; Gaffo et al., 2013), animal (Kang et al., 2005; Corry et al., 2008) studies, and clinical trials (Feig et al., 2008) suggested that SUA might increase blood pressure (BP). Some studies indicated that hypertension might mediate the effect of HUA on stroke (Hozawa et al., 2006; Shi et al., 2017; Chaudhary et al., 2020).

China bears a huge burden of hypertension and stroke (Wang et al., 2018). Accumulating evidence suggests that hypertension plays a definitive role in the development of atherosclerosis and is the primary cause of stroke (Buonacera et al., 2019). Considering the controversial role of HUA as an independent risk factor for stroke events in the Chinese hypertensive patients (Chien et al., 2005; Bos et al., 2006; Shi et al., 2017; Zhang et al., 2020), we investigated the relationship between HUA and the risk of first stroke incidence in the Chinese adults with hypertension.

## Materials and Methods

### Study Design and Participants

Data analyzed in this study were the baseline of the ongoing China H-type Hypertension Registry Study (registration number: ChiCTR1800017274). The data collection approaches and the established standards of inclusion or exclusion have been described previously (Li M. et al., 2020). Briefly, the study is a real-world, multicenter, observational study, conducted in March 2018 at Wuyuan, Jiangxi province of China. The enrolled population was hypertensive patients aged 18 years and older. The exclusion criteria included psychological or nervous system impairment resulting in an inability to demonstrate informed consent, unable to be a long-term follow-up according to the study protocol or plans to relocate in the near future. The study was conducted in accordance with the Declaration of Helsinki, and the protocol was approved by the Ethics Committee of the Institute of Biomedicine, Anhui Medical University (no. CH1059). All participants provided written informed consent.

**FIGURE 1:**
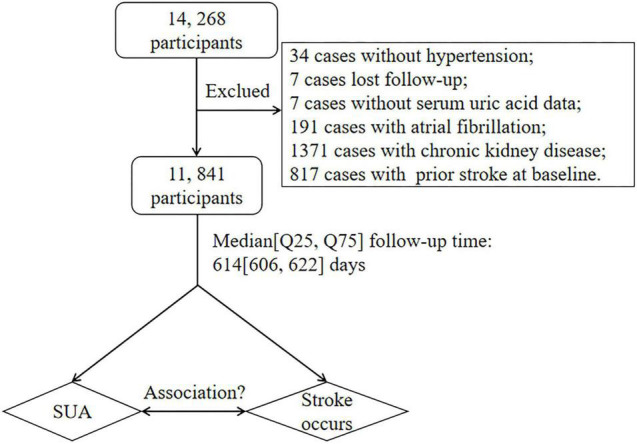
Data flow chart of participants in our analysis. SUA, serum uric acid; eGFR, estimated glomerular filtration rate.

In total, 14,268 participants completed the baseline investigation between March 2018 and August 2018. We followed these participants between August 31, 2018 and March 31, 2020. The median follow-up duration was 614 (606, 622) days. After excluding 34 individuals without hypertension at baseline, 7 cases were lost to follow-up, 7 cases were without SUA data, 191 cases were with atrial fibrillation, 1,371 cases were with estimated glomerular filtration rate (eGFR) ≤ 60 ml/min/1.73 m^2^, and 817 cases were with prior stroke at baseline, finally 11, 841 participants were included in our analysis (Figure 1).

### Clinical Data Collection at Baseline

Demographic characteristics (age and sex), lifestyle factors (smoking and drinking status), medical history [hypertension, DM, dyslipidemia, atrial fibrillation, cerebral stroke (ischemic stroke, intracerebral hemorrhage, or unspecified stroke), coronary heart disease (CHD) and chronic kidney disease (CKD)], and medication usage were gathered by professional researchers through questionnaire survey. A person who smoked at least one cigarette a day was classified as a smoker. A person who drank > 20 g of ethanol a day was considered a drinker. Atrial fibrillation was diagnosed based on a medical history and through resting supine standard 12-lead surface ECG.

Anthropometric measurements for each patient, such as weight, height, waistline, hipline, systolic blood pressure (SBP), diastolic blood pressure (DBP), and heart rate (HR), were obtained by researchers. Due to the health-screening protocols, single-point measurements of anthropometric indicators were obtained. Waistline and hipline were measured using an inelastic measuring tape with 0.1 cm resolution. BP was measured, with the participant in a sitting position using the Omron HBP-1,300 Professional Portable Blood Pressure Monitor (Kyoto, Japan) on the right arm, which was supported at the heart level. After a 5-min rest period, BP was measured four times, and SBP and DBP were calculated as the average of the last three readings. Body mass index (BMI) was calculated as the body weight in kilograms divided by the square of the height in meters (kg/m^2^). Waist hip rate (WHR) was calculated as the waistline in centimeters divided by the hipline in centimeters. In our study, subjects were divided into three categories according to BMI levels (<24 kg/m^2^, control group; 24–28 kg/m^2^, overweight; and ≥ 28 kg/m^2^, general obesity) (Liu et al., 2019). Central obesity was defined as WHR ≥ 0.9 for males and ≥ 0.85 for women (Liu et al., 2019).

### Laboratory Assay

Blood samples were collected utilizing venipuncture after an overnight fast of at least 12 h. The levels of plasma total homocysteine (Hcy), fasting blood glucose (FBG), total cholesterol (TC), total triglyceride (TG), high-density lipoprotein cholesterol (HDL-C), low-density lipoprotein cholesterol (LDL-C), SUA, blood urea nitrogen (BUN), and serum creatinine, total and direct bilirubin, aspartate aminotransferase (AST), and alanine aminotransferase (ALT) were measured using automatic clinical analyzers (Beckman Coulter, Brea, CA, United States). In our analysis, diagnosis of DM was defined as fasting glucose > 7.0 mmol/l and/or self-reported diabetes (Jia et al., 2019). Dyslipidemia was defined as having one or more of the following features: elevated TG (≥2.3 mmol/L), elevated TC (≥6.2 mmol/L), elevated LDL-C (≥4.1 mmol/L), reduced HDL-C (<1.0 mmol/L), or on appropriate lipid-lowering medication (Joint Committee for Developing Chinese guidelines on Prevention and Treatment of Dyslipidemia in Adults, 2007). HUA was defined as an SUA > 420 μmol/L in men and > 360 μmol/L in women among the Chinese population (Tu et al., 2019; Chen et al., 2020). The eGFR was calculated by the equation of CKD Epidemiology Collaboration (CKD-EPI) (Levey et al., 2009).

### Primary Outcome and Definitions

CT or MRI was conducted to confirm first stroke events, which was defined as a sudden or rapid onset of a focal neurological deficit lasting more than 24 h or until death. According to the International Classification of Diseases, 10th edition (ICD-10), strokes were coded as I61 (hemorrhagic stroke), I63 (ischemic stroke), or I64 (unspecified stroke), and the patients were blinded to the baseline information collected by trained staff. If the patients experienced an in-hospital stroke event, facility and physician records were referred to for stroke diagnosis. In the event that a stroke occurred outside a hospital, the medical records of the patient, along with the accounts provided by the family members, were used, allowing the experts to reach a consensus regarding the diagnosis of a stroke following a comprehensive consideration, such as the history, recent condition, and symptoms before and after the event. Causes of death included stroke, heart disease, cancer, and respiratory diseases. All the death mortalities were ascertained from the Local Healthcare Security Administration, Centers for Disease Control and Prevention, and hospitals.

### Statistical Analysis

Continuous variables are presented as the means ± SD or the median (quartiles), as appropriate, and are compared using the Student’s *t*-test, one-way analysis, or the Mann-Whitney *U*-test, depending on whether the quantitative data were consistent with a normal distribution. Categorical variables were presented as count (percentage), differences between groups were measured by chi-square test.

Secondly, to address the linearity or not between continuous SUA levels and the risk of the first stroke, a Cox proportional hazards ratio (HR) model with cubic spline functions and smooth curve fitting (restricted cubic spline method) was performed (Supplementary Figure 1). We used three different Cox proportional hazards models to examine the association between SUA levels and the risk of first stroke. The crude model was not adjusted for any confounder. The model I was adjusted for age, sex, SBP, DBP, and HR. The model II was a confounder model. The confounder model screened covariates, such as age, sex, SBP, DBP, HR, BMI, WHR, smoking and drinking status, homocysteine, TC, TG, HDL-C, LDL-C, AST, ALT, eGFR, total and direct bilirubin, DM, antihypertensive agents, lipid-lowering agents, and antiplatelet agents except for the independent variable itself. We selected these confounders on the basis of their associations with the outcomes of interest or a change in effect estimate of more than 10% when added to this model. Supplementary Table 1 shows the association of each confounder with stroke occurs. Linear trend tests were realized by entering a median value of each category of SUA levels as a continuous variable.

Furthermore, the effects of HUA on first stroke events were evaluated with the use of Kaplan-Meier curves (log-rank test). Finally, subgroup analysis was executed by stratified and interaction test to investigate the robustness between SUA levels and the risk of the first stroke. In consideration of that, there was a threshold effect of age on SUA levels, the generalized additive model and smooth curve fitting (penalized spline method) were used to visually show the relationship between age and SUA levels. If non-linearity was detected, we first use a recursive algorithm to calculate the inflection points and then construct a two-segment binary logistic regression model on both sides of the inflection points.

All statistical analyses were performed using the statistical package R (The R Foundation, version 3.4.3)^1^ and the Empower^2^ (R; X&Y Solutions, Inc., Boston, MA, United States). All *P*-values are two-tailed, and *P* < 0.05 was considered statistically significant.

## Results

### Patient Characteristics at Baseline

The present study included 11, 841 Chinese adult hypertensive individuals (age: 62.95 ± 9.14 years, range 27–93 years; men, 45.67%), and the prevalence of HUA was 52.23%. The clinical characteristics of the study participants grouped by SUA quartiles are presented in Table 1. Compared with patients with SUA concentrations ≤ 325.80 μmol/L, there was a higher proportion of men, overweight, general obesity, central obesity, smoking and drinking habits, CHD, DM, dyslipidemia and antihypertensive agent usage, elevated values of DBP, BMI, WHR, Hcy, TC, TG, LDL-C, BUN, serum creatinine, AST, ALT, total and direct bilirubin, and lower values of SBP, HDL-C, and eGFR in patients of the third and highest SUA quartiles (all *P*-values < 0.05, Table 1). The clinical characteristics of participants grouped by age or sex are also assessed in Supplementary Table 2. Subjects aged less than 60 years had a higher prevalence of overweight and general obesity, central obesity, HUA, DM, and dyslipidemia, a lower prevalence of smoking habits, CHD, antihypertensive and antiplatelet agent usage, and a lower level of eGFR than the aging group (all *P*-values < 0.05, Supplementary Table 2).

**TABLE 1:** Baseline characteristics of the study population.

Characteristics	Total subjects	Quartiles of serum uric acid levels (μ /L)				*P*-value
		Q1 [38.00, 325.80]	Q2 [326.00, 393.30]	Q3 [394.00, 472.00]	Q4 [473.00, 1056.00]	
Number of subjects (n)	11,841	2,960	2,936	2,967	2,978	
Age (years)	62.95 ± 9.14	62.74 ± 8.74	63.67 ± 8.85	63.22 ± 9.18	62.19 ± 9.71	< 0.001
Male, n (%)	5,408 (45.67%)	572 (19.32%)	1,109 (37.77%)	1,629 (54.90%)	2,098 (70.45%)	< 0.001
SBP (mmHg)	148.52 ± 17.37	150.44 ± 16.91	149.37 ± 17.31	148.12 ± 17.55	146.18 ± 17.41	< 0.001
DBP (mmHg)	89.45 ± 10.51	88.90 ± 10.22	88.75 ± 10.21	89.65 ± 10.67	90.48 ± 10.84	< 0.001
MAP (mmHg)	109.14 ± 11.07	109.42 ± 10.84	108.96 ± 10.90	109.14 ± 11.27	109.05 ± 11.25	0.440
HR (times/min)	76.56 ± 13.84	77.57 ± 14.48	76.41 ± 13.41	75.97 ± 13.24	76.28 ± 14.15	< 0.001
BMI (kg/m^2^)	23.71 ± 3.54	23.00 ± 3.46	23.43 ± 3.52	23.87 ± 3.60	24.51 ± 3.54	< 0.001
**BMI group (kg/m^2^)**						< 0.001
Control (<24)	6,521 (55.10%)	1,906 (64.41%)	1,731 (59.04%)	1,547 (52.16%)	1,337 (44.90%)	
Overweight (≥24, < 28)	4,041 (34.14%)	850 (28.73%)	935 (31.89%)	1,069 (36.04%)	1,187 (39.86%)	
General obesity (≥28)	1,273 (10.76%)	203 (6.86%)	266 (9.07%)	350 (11.80%)	454 (15.25%)	
WHR	0.91 ± 0.17	0.90 ± 0.07	0.91 ± 0.31	0.92 ± 0.07	0.93 ± 0.07	< 0.001
Central obesity, n (%)	8,422 (71.13%)	2,065 (69.76%)	2,045 (69.65%)	2,082 (70.17%)	2,230 (74.88%)	< 0.001
**Smoking status, n (%)**						
Never	7,035 (59.42%)	2,299 (77.67%)	1,891 (64.45%)	1,537 (51.80%)	1,308 (43.92%)	< 0.001
Former	1,754 (14.82%)	222 (7.50%)	369 (12.58%)	517 (17.43%)	646 (21.69%)	
Current	3,050 (25.76%)	439 (14.83%)	674 (22.97%)	913 (30.77%)	1,024 (34.39%)	
**Drinking status, n (%)**						
Never	7,611 (64.29%)	2,298 (77.64%)	2,026 (69.05%)	1,761 (59.35%)	1,526 (51.24%)	< 0.001
Former	1,481 (12.51%)	343 (11.59%)	356 (12.13%)	400 (13.48%)	382 (12.83%)	
Current	2,747 (23.20%)	319 (10.78%)	552 (18.81%)	806 (27.17%)	1,070 (35.93%)	
Hcy (μmol/L)	14.47 (12.25–17.95)	13.11 (11.41–16.00)	14.01 (11.99–16.95)	14.86 (12.58–18.34)	16.02 (13.42–20.30)	< 0.001
FBG (mmol/L)	6.17 ± 1.61	6.13 ± 1.75	6.18 ± 1.76	6.15 ± 1.46	6.23 ± 1.43	0.132
TC (mmol/L)	5.19 ± 1.09	5.10 ± 1.03	5.17 ± 1.08	5.19 ± 1.11	5.29 ± 1.13	< 0.001
TG (mmol/L)	1.47 (1.04–2.17)	1.32 (0.98–1.85)	1.42 (1.00–2.04)	1.52 (1.06–2.22)	1.74 (1.17–2.62)	< 0.001
HDL-C (mmol/L)	1.58 ± 0.43	1.64 ± 0.43	1.60 ± 0.42	1.55 ± 0.42	1.53 ± 0.43	< 0.001
LDL-C (mmol/L)	3.00 ± 0.80	2.93 ± 0.75	2.99 ± 0.81	3.02 ± 0.81	3.09 ± 0.82	< 0.001
SUA (**μ** mol/L)	406.26 ± 113.31	275.18 ± 39.35	359.72 ± 19.25	430.54 ± 22.70	558.23 ± 75.72	< 0.001
HUA, n (%)	6,184 (52.23%)	0 (0.00%)	820 (27.93%)	2,386 (80.42%)	2,978 (100.00%)	< 0.001
BUN (mmol/L)	5.23 ± 1.45	5.02 ± 1.41	5.20 ± 1.41	5.28 ± 1.44	5.43 ± 1.50	< 0.001
Serum creatinine (mmol/L)	64.38 ± 16.94	52.97 ± 13.61	60.86 ± 14.16	67.46 ± 14.91	76.12 ± 15.78	< 0.001
eGFR (ml/min/1.73 m^2^)	93.21 ± 14.36	99.76 ± 13.11	94.71 ± 13.00	91.57 ± 13.71	86.84 ± 14.40	< 0.001
Human serum albumin (g/L)	46.82 ± 4.00	46.53 ± 4.08	46.76 ± 3.99	46.85 ± 3.94	47.14 ± 3.98	< 0.001
Total bilirubin (mmol/L)	13.40 (10.40–17.50)	12.60 (9.88–16.20)	13.20 (10.20–17.40)	13.70 (10.60–17.80)	14.20 (10.90–18.60)	< 0.001
Direct bilirubin (mmol/L)	5.20 (4.10–6.50)	4.90 (4.00–6.20)	5.10 (4.10–6.50)	5.20 (4.20-6.50)	5.40 (4.30–6.90	< 0.001
AST (U/L)	24.00 (20.00–30.00)	22.50 (19.00–27.00)	24.00 (20.00–29.00)	25.00 (21.00–31.00)	26.00 (22.00–33.00)	< 0.001
ALT (U/L)	17.00 (13.00–24.00)	14.00 (11.00–20.00)	16.00 (12.00–23.00)	18.00 (13.00–25.00)	20.00 (14.00–28.00)	< 0.001
CHD, n (%)	538 (4.54%)	113 (3.82%)	122 (4.16%)	151 (5.09%)	152 (5.10%)	0.032
DM, n (%)	2,064 (17.43%)	462 (15.61%)	485 (16.52%)	544 (18.34%)	573 (19.24%)	< 0.001
Dyslipidemia, n (%)	4,402 (37.18%)	877 (29.63%)	1,001 (34.09%)	1,139 (38.39%)	1,385 (46.51%)	< 0.001
Antihypertensive agents, n (%)	7,366 (62.22%)	1,760 (59.46%)	1,823 (62.13%)	1,871 (63.06%)	1,912 (64.20%)	0.001
Hypoglycemic agents, n (%)	554 (4.68%)	150 (5.07%)	149 (5.07%)	129 (4.35%)	126 (4.23%)	0.250
Lipid-lowering agents, n (%)	285 (2.41%)	81 (2.74%)	63 (2.15%)	74 (2.49%)	67 (2.25%)	0.452
Antiplatelet agents, n (%)	265 (2.24%)	57 (1.93%)	63 (2.15%)	79 (2.66%)	66 (2.22%)	0.274

*SBP, systolic blood pressure; DBP, diastolic blood pressure; MAP, mean arterial pressure; HR, heart rate; BMI, body mass index; WHR, waist hip rate; Hcy, homocysteine; FBG, fasting blood glucose; TC, total cholesterol; TG, total triglyceride; HDL-C, high-density lipoprotein cholesterol; LDL-C, low-density lipoprotein cholesterol; SUA, serum uric acid; HUA, hyperuricemia; BUN, blood urea nitrogen; eGFR, estimated glomerular filtration rate; AST, aspartate aminotransferase; ALT, alanine aminotransferase; CHD, coronary heart disease; DM, diabetes mellitus.*

### Cumulative Incidence of Incident Stroke

Of the study population, a total of 99 (0.84%) first stroke events (51 ischemic events, 15 hemorrhagic events, and 33 unspecified stroke events) occurred during a median 614-day follow-up period (Table 2). The average time was 612.14 ± 32.12 days from the baseline to the first stroke. There were no significant differences in stroke events groups by SUA quartiles (*P* > 0.05; Table 2). There was no difference in the causes of death between the groups (Table 2).

**TABLE 2:** Occurrence of follow-up events of the study population.

Characteristics	Total subjects	Quartiles of serum uric acid levels (μ mol/L)				*P*-value
		Q1 [38.00, 325.80]	Q2 [326.00, 393.30]	Q3 [394.00, 472.00]	Q4 [473.00, 1056.00]	
Incident stroke, n (%)	99 (0.84%)	22 (0.74%)	23 (0.78%)	29 (0.98%)	25 (0.84%)	0.772
**Stroke subtypes, n (%)**						0.859
Ischemic stroke	51 (0.43%)	10 (0.34%)	14 (0.48%)	12 (0.40%)	15 (0.50%)	
Hemorrhagic stroke	15 (0.13%)	3 (0.10%)	3 (0.10%)	6 (0.20%)	3 (0.10%)	
Unspecified stroke	33 (0.28%)	9 (0.30%)	6 (0.20%)	11 (0.37%)	7 (0.24%)	
**Causes of death, n (%)**						0.310
Stroke	34 (0.29%)	9 (0.30%)	8 (0.27%)	10 (0.34%)	7 (0.24%)	
Heart disease	29 (0.24%)	5 (0.17%)	2 (0.07%)	12 (0.40%)	10 (0.34%)	
Cancer	40 (0.34%)	13 (0.44%)	9 (0.31%)	9 (0.30%)	9 (0.30%)	
Respiratory diseases	4 (0.03%)	0 (0.00%)	1 (0.03%)	1 (0.03%)	2 (0.07%)	
Others	17 (0.14%)	2 (0.07%)	2 (0.07%)	6 (0.20%)	7(0.24%)	

### Association Between Serum Uric Acid Levels and First Stroke Events

There were no significant differences in the SUA levels and proportion of HUA between non-stroke patients and patients with first stroke (*P* > 0.05; Supplementary Table 3). Cox proportional hazards models indicated that SUA levels were not associated with the risk of first stroke events (adjusted-HR per SD increase: 0.98, 95% CI 0.76–1.26, *P* = 0.889, Table 3), ischemic stroke events (adjusted-HR per SD increase: 0.97, 95% CI 0.69–1.37, *P* = 0.871) or hemorrhagic stroke events (adjusted-HR per SD increase: 0.73, 95% CI 0.36–1.48, *P* = 0.387). In comparison to the group without HUA, there was also no significantly higher risk of total first stroke events (adjusted-HR: 1.22, 95% CI 0.79–1.90, *P* = 0.373, Table 3), ischemic stroke events (adjusted-HR: 1.28, 95% CI 0.69–2.39, *P* = 0.440), or hemorrhagic stroke events (adjusted-HR: 1.48, 95% CI 0.45–4.85, *P* = 0.516) in the population with HUA. Furthermore, compared with patients with SUA concentrations ≤ 325.80 μmol/L, there was no significantly higher risk of total first stroke events for patients in the third and highest SUA quartiles (adjusted-HR: 0.92, 95% CI 0.50–1.70, *P* = 0.786; adjusted-HR: 0.72, 95% CI 0.36–1.44, *P* = 0.354, respectively; *P* for trend = 0.391, Table 3), ischemic stroke events (adjusted-HR: 0.73, 95% CI 0.29–1.80, *P* = 0.493; adjusted-HR: 0.77, 95% CI 0.30–1.99, *P* = 0.585, respectively; *P* for trend = 0.498) or hemorrhagic stroke events (adjusted-HR: 1.26, 95% CI 0.25–6.38, *P* = 0.782; adjusted-HR: 0.53, 95% CI 0.08–3.73, *P* = 0.527, respectively; *P* for trend = 0.397).

**TABLE 3:** Hazard ratios of serum uric acid level categories for first stroke events.

Variables	Event, n (%)	Crude model		Model I		Model II	
		HR (95%CI)	*P-*value	HR (95%CI)	*P*-value	HR (95%CI)	*P*-value
**Total stroke**							
Per *SD* μmol/L increase	99 (0.84%)	1.10 (0.91, 1.34)	0.308	1.05 (0.84, 1.30)	0.669	0.98 (0.76, 1.26)	0.889^a^
**HUA**							
No	40 (0.71%)	Ref		Ref		Ref	
Yes	59 (0.95%)	1.35 (0.90, 2.02)	0.142	1.31 (0.87, 1.97)	0.195	1.22 (0.79, 1.90)	0.373^b^
**Quartiles of SUA**							
Q1 [38.00, 325.80]	22 (0.74%)	Ref		Ref		Ref	
Q2 [326.00, 393.30]	23 (0.78%)	1.05 (0.59, 1.90)	0.860	0.93 (0.51, 1.69)	0.815	0.85 (0.46, 1.56)	0.602^c^
Q3 [394.00, 472.00]	29 (0.98%)	1.32 (0.76, 2.30)	0.331	1.08 (0.60, 1.94)	0.786	0.92 (0.50, 1.70)	0.786^c^
Q4 [473.00, 1056.00]	25 (0.84%)	1.13 (0.64, 2.01)	0.676	0.93 (0.50, 1.72)	0.815	0.72 (0.36, 1.44)	0.354^c^
P for trend		0.576		0.896		0.391	
**Ischemic stroke**							
Per *SD* μmol/L increase	51 (0.43%)	1.18 (0.91, 1.52)	0.225	1.18 (0.88, 1.58)	0.265	0.97 (0.69, 1.37)	0.871^d^
**HUA**							
No	18 (0.32%)	Ref		Ref		Ref	
Yes	33 (0.53%)	1.68 (0.95, 2.99)	0.077	1.67 (0.94, 2.98)	0.083	1.28 (0.69, 2.39)	0.440^e^
**Quartiles of SUA**							
Q1 [38.00, 325.80]	10 (0.34%)	Ref		Ref		Ref	
Q2 [326.00, 393.30]	14 (0.48%)	1.41 (0.63, 3.19)	0.404	1.28 (0.56, 2.90)	0.563	1.01 (0.44, 2.34)	0.975^f^
Q3 [394.00, 472.00]	12 (0.40%)	1.20 (0.52, 2.78)	0.674	1.06 (0.44, 2.53)	0.894	0.73 (0.29, 1.80)	0.493^f^
Q4 [473.00, 1056.00]	15 (0.50%)	1.49 (0.67, 3.33)	0.327	1.39 (0.59, 3.26)	0.454	0.77 (0.30, 1.99)	0.585^f^
P for trend		0.417		0.535		0.498	
**Hemorrhagic stroke**							
Per *SD* μmol/L increase	15 (0.13%)	0.99 (0.59, 1.64)	0.962	0.68 (0.38, 1.22)	0.197	0.73 (0.36, 1.48)	0.387^g^
**HUA**							
No	6 (0.11%)	Ref		Ref		Ref	
Yes	9 (0.15%)	1.37 (0.49, 3.86)	0.548	1.06 (0.37, 3.06)	0.911	1.48 (0.45, 4.85)	0.516^h^
**Quartiles of SUA**							
Q1 [38.00, 325.80]	3 (0.10%)	Ref		Ref		Ref	
Q2 [326.00, 393.30]	3 (0.10%)	1.01 (0.20, 5.00)	0.992	0.75 (0.14, 3.92)	0.735	0.79 (0.14, 4.49)	0.788^i^
Q3 [394.00, 472.00]	6 (0.20%)	2.00 (0.50, 7.99)	0.328	1.09 (0.24, 4.93)	0.911	1.26 (0.25, 6.38)	0.782^i^
Q4 [473.00, 1056.00]	3 (0.10%)	0.99 (0.20, 4.93)	0.994	0.40 (0.07, 2.33)	0.310	0.53 (0.08, 3.73)	0.527^i^
P for trend		0.962		0.197		0.397	

*HUA, hyperuricemia; Ref, reference; HR, hazard ratio; CI, confidence interval; SD, standard deviation.*

*Model I adjusted for age, sex, SBP, DBP, and HR.*

*Model II: ^a^Adjusted for age, sex, SBP, DBP, HR, BMI, WHR, smoking and drinking status, Hcy, TG, HDL-C, LDL-C, eGFR, total bilirubin, ALT, DM, and antiplatelet agents. ^b^Adjusted for age, sex, SBP, DBP, HR, BMI, smoking status, Hcy, TG, eGFR, ALT, and antiplatelet agents. ^c^Adjusted for age, sex, SBP, DBP, HR, BMI, WHR, smoking and drinking status, Hcy, TG, HDL-C, LDL-C, eGFR, total bilirubin, AST, ALT, and antiplatelet agents. ^d^Adjusted for age, sex, SBP, DBP, BMI, WHR, smoking and drinking status, Hcy, TG, LDL-C, eGFR, AST, ALT, DM, antihypertensive agents, and lipid-lowering agents. ^e^Adjusted for age, SBP, BMI, drinking status, Hcy, TG, LDL-C, eGFR, and ALT. ^f^Adjusted for age, sex, SBP, BMI, smoking and drinking status, Hcy, TG, HDL-C, LDL-C, eGFR, AST, ALT, DM, antihypertensive agents, and lipid-lowering agents. ^g^Adjusted for age, sex, SBP, DBP, HR, BMI, WHR, smoking and drinking status, Hcy, TG, HDL-C, LDL-C, eGFR, total bilirubin, AST, ALT, DM, antihypertensive agents, and antiplatelet agents. ^h^Adjusted for age, sex, SBP, DBP, HR, WHR, smoking and drinking status, Hcy, TG, HDL-C, LDL-C, eGFR, AST, and antiplatelet agents. ^i^Adjusted for age, sex, SBP, DBP, HR, BMI, WHR, smoking and drinking status, Hcy, TG, HDL-C, LDL-C, eGFR, total bilirubin, AST, ALT, DM, antihypertensive agents, lipid-lowering agents, and antiplatelet agents.*

**FIGURE 2:**
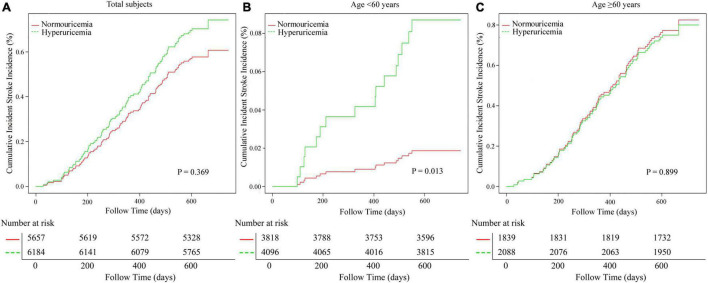
The cumulative total first stroke risks in the study population. **(A)** Adjusted for age, sex, SBP, DBP, HR, BMI, smoking status, Hcy, TG, eGFR, ALT, and antiplatelet agents. **(B)** Adjusted for SBP, DBP, HR, WHR, TG, LDL-C, eGFR, ALT, lipid-lowering agents, and antiplatelet agents. **(C)** Adjusted for sex, SBP, DBP, HR, BMI, smoking and drinking status, Hcy, TG, HDL-C, LDL-C, AST, eGFR, and antiplatelet agents. eGFR, estimated glomerular filtration rate; SBP, systolic blood pressure; DBP, diastolic blood pressure; HR, heart rate, BMI, body mass index; Hcy, homocysteine; TG, total triglyceride; HDL-C, high-density lipoprotein cholesterol; LDL-C, low-density lipoprotein cholesterol; AST, aspartate aminotransferase; ALT, alanine aminotransferase.

Restricted cubic spline indicated that SUA levels were not associated with the risk of first stroke events (*P*-value of log-likelihood ratio test = 0.138, Supplementary Figure 1). Survival analysis further confirmed this irrelevant association of HUA with first stroke events (Kaplan–Meier, log-rank *P* = 0.369; Figure 2A).

### Subgroup Analyses by Potential Effect Modifiers

To explore whether this irrelevant association between SUA levels and first stroke events was still stable in different subgroups, we conducted stratified and interaction analyses.

We used the generalized additive model and penalized spline method to assess that whether there was a non-linear relationship between age and SUA levels (Supplementary Figure 4). In the adjusted smoothing curve, the relationship between age and SUA levels was not linear. With the increase of age, the SUA levels decreased first and then leveled off. Visual inspection shows that the inflection point is around 57 years old. We further fitted the association between age and SUA levels using the two-piecewise binary logistic regression model. The inflection point of age was 57 (Supplementary Table 4). Effect size [β (95% CI)] of age on SUA levels was −2.97 (−3.59, −2.35) on the left side and −1.67 (−1.99, −1.35) on the right of the inflection point. These results suggested that there was a threshold effect of age on SUA levels.

In consequence, we chose the age of 60 for stratification and sub-analysis. We found that this association between SUA levels and first stroke events was modified by aging (Table 4). In a group with ages of less than 60 years, subjects with HUA had a significantly higher risk of first stroke events compared to the population without HUA (adjusted-HR: 4.89, 95% CI 1.36−17.63, *P* = 0.015). However, this positive association was disappeared in aging population (adjusted-HR: 0.97, 95% CI 0.60−1.56, *P* = 0.886; *P*-value for interaction = 0.043). Survival analysis further confirmed this discrepancy (Kaplan–Meier, log-rank *P* = 0.013 for the non-aging group, *P* = 0.899 for aging group, respectively; Figures 2B,C).

**TABLE 4:** Hazard ratios of serum uric acid level categories for total first stroke events by age in different models.

Variables	Event, n (%)	Crude model		Model I		Model II		*P*-value for interaction
		HR (95%CI)	*P*-value	HR (95%CI)	*P*-value	HR (95%CI)	*P*-value	
*Age < 60 years*								
**SUA**								
Per *SD* μmol/L increase	16 (0.41%)	1.19 (0.77, 1.84)	0.443	1.06 (0.63, 1.77)	0.837	1.75 (0.95, 3.20)	0.072^a^	
**HUA**								
No	5 (0.27%)	Ref		Ref		Ref		
Yes	11 (0.53%)	1.94 (0.67, 5.60)	0.219	1.84 (0.61, 5.56)	0.280	4.89 (1.36, 17.63)	0.015^b^	
**Quartiles of SUA**								
Q1 [93.00, 325.80]	3 (0.30%)	Ref		Ref		Ref		
Q2 [326.00, 393.30]	5 (0.56%)	1.89 (0.45, 7.91)	0.386	1.40 (0.31, 6.24)	0.658	1.84 (0.33, 10.22)	0.486^c^	
Q3 [394.00, 472.00]	4 (0.42%)	1.42 (0.32, 6.36)	0.647	0.89 (0.18, 4.57)	0.893	1.55 (0.25, 9.62)	0.639^c^	
Q4 [473.00, 1056.00]	4 (0.38%)	1.27 (0.28, 5.67)	0.757	0.79 (0.14, 4.29)	0.783	2.47 (0.33, 18.65)	0.382^c^	
P for trend		0.931		0.619		0.442		
*Age ≥ 60 years*								
**SUA**								
Per *SD* μmol/L increase	83 (1.05%)	1.10 (0.89, 1.37)	0.387	1.02 (0.81, 1.29)	0.868	0.86 (0.65, 1.13)	0.284^d^	0.042^g^
**HUA**								
No	35 (0.92%)	Ref		Ref		Ref		0.043^h^
Yes	48 (1.17%)	1.28 (0.83, 1.99)	0.267	1.24 (0.80, 1.92)	0.341	0.97 (0.60, 1.56)	0.886^e^	
**Quartiles of SUA**								
Q1 [38.00, 325.00]	19 (0.98%)	Ref		Ref		Ref		0.482^i^
Q2 [326.00, 393.00]	18 (0.88%)	0.90 (0.47, 1.73)	0.761	0.85 (0.44, 1.63)	0.620	0.70 (0.36, 1.36)	0.287^f^	
Q3 [394.00, 472.00]	25 (1.24%)	1.28 (0.70, 2.32)	0.427	1.11 (0.59, 2.08)	0.747	0.78 (0.40, 1.51)	0.454^f^	
Q4 [473.00, 879.00]	21 (1.10%)	1.13 (0.60, 2.10)	0.706	0.92 (0.47, 1.79)	0.808	0.55 (0.26, 1.17)	0.120^f^	
P for trend		0.515		0.967		0.163		

*SUA, serum uric acid; HUA, hyperuricemia; Ref, reference; HR, hazard ratio; CI, confidence interval; SD, standard deviation.*

*Model I adjusted for sex, SBP, DBP, and HR.*

*Model II: ^a^Adjusted for sex, SBP, DBP, HR, BMI, WHR, Hcy, TG, LDL-C, eGFR, total bilirubin, AST, ALT, lipid-lowering agents and antiplatelet agents. ^b^Adjusted for SBP, DBP, HR, WHR, TG, LDL-C, eGFR, ALT, lipid-lowering agents, and antiplatelet agents. ^c^Adjusted for sex, SBP, DBP, BMI, WHR, smoking and drinking status, Hcy, TG, HDL-C, LDL-C, eGFR, total bilirubin, AST, ALT, DM, antihypertensive agents, lipid-lowering agents, and antiplatelet agents. ^d^Adjusted for sex, SBP, DBP, HR, BMI, smoking and drinking status, Hcy, TG, HDL-C, LDL-C, and eGFR. ^e^Adjusted for sex, SBP, DBP, HR, BMI, smoking and drinking status, Hcy, TG, HDL-C, LDL-C, AST, eGFR and antiplatelet agents. ^f^Adjusted for sex, SBP, DBP, HR, BMI, WHR, smoking and drinking status, Hcy, TG, HDL-C, LDL-C, eGFR, and AST. ^g^Adjusted for sex, SBP, DBP, HR, BMI, WHR, smoking and drinking status, Hcy, TG, HDL-C, LDL-C, eGFR, total bilirubin, ALT, DM, antiplatelet agents and the interaction terms for following variables: sex, SBP, DBP, HR, BMI, WHR, smoking and drinking status, Hcy, TG, HDL-C, LDL-C, eGFR, total bilirubin, ALT, DM, antiplatelet agents. ^h^Adjusted for sex, SBP, DBP, HR, BMI, smoking status, Hcy, TG, eGFR, ALT, antiplatelet agents and the interaction terms for following variables: SBP, DBP, HR, Hcy, TG, eGFR, ALT, and antiplatelet agents. ^i^Adjusted for sex, SBP, DBP, HR, BMI, WHR, smoking and drinking status, Hcy, TG, HDL-C, LDL-C, eGFR, total bilirubin, ALT, AST, antiplatelet agents and the interaction terms for following variables: age, SBP, DBP, HR, BMI, WHR, smoking and drinking status, Hcy, TG, HDL-C, LDL-C, eGFR, total bilirubin, ALT, AST, antiplatelet agents.*

However, subgroup analyses showed that there were not significant interactions in any of the subgroups, such as sex (men vs. women, Table 5 and Supplementary Figure 2), mean arterial pressure tertiles (Table 6 and Supplementary Figure 3), SBP tertiles (Supplementary Table 5), DBP tertiles (Supplementary Table 6), antihypertensive drugs usage (Table 7), body mass index tertiles (Supplementary Table 7), and central obesity (Supplementary Table 8).

**TABLE 5:** Hazard ratios of serum uric acid level categories for total first stroke events by sex in different models.

Variables	Event, n (%)	Crude model		Model I		Model II		*P*-value for interaction
		*HR (95%CI)*	*P*-value	HR (95%CI)	*P*-value	HR (95%CI)	*P*-value	
*Males*								
**SUA**								
Per *SD* μmol/L increase	56 (1.04%)	1.02 (0.78, 1.33)	0.905	1.05 (0.80, 1.39)	0.718	1.00 (0.72, 1.39)	0.987^a^	
**HUA**								
No	22 (0.97%)	Ref		Ref		Ref		
Yes	34 (1.08%)	1.11 (0.65, 1.91)	0.699	1.14 (0.66, 1.95)	0.646	1.06 (0.58, 1.91)	0.856^b^	
**Quartiles of SUA**								
Q1 [38.00, 325.00]	8 (1.40%)	Ref		Ref		Ref		
Q2 [326.00, 393.00]	9 (0.81%)	0.58 (0.22, 1.50)	0.260	0.56 (0.21, 1.46)	0.233	0.50 (0.19, 1.31)	0.159^c^	
Q3 [394.00, 472.00]	19 (1.17%)	0.83 (0.36, 1.91)	0.665	0.80 (0.35, 1.85)	0.605	0.67 (0.28, 1.58)	0.356^c^	
Q4 [473.00, 1056.00]	20 (0.95%)	0.68 (0.30, 1.55)	0.357	0.71 (0.31, 1.62)	0.411	0.54 (0.22, 1.35)	0.187^c^	
P for trend		0.576		0.494		0.753		
*Females*								
**SUA**								
Per *SD* μmol/L increase	43 (0.67%)	1.03 (0.73, 1.45)	0.864	1.02 (0.72, 1.45)	0.892	0.98 (0.66, 1.47)	0.922^d^	1.000^g^
**HUA**								
No	18 (0.53%)	Ref		Ref		Ref		0.274^h^
Yes	25 (0.82%)	1.56 (0.85, 2.86)	0.154	1.54 (0.84, 2.85)	0.165	1.70 (0.87, 3.30)	0.118^e^	
**Quartiles of SUA**								
Q1 [108.00, 325.80]	14 (0.59%)	Ref		Ref		Ref		0.539^i^
Q2 [326.00, 393.00]	14 (0.77%)	1.31 (0.62, 2.75)	0.477	1.27 (0.60, 2.69)	0.529	1.30 (0.60, 2.82)	0.513^f^	
Q3 [394.00, 472.00]	10 (0.75%)	1.28 (0.57, 2.88)	0.556	1.28 (0.56, 2.91)	0.557	1.21 (0.50, 2.94)	0.675^f^	
Q4 [473.00, 879.00]	5 (0.57%)	0.97 (0.35, 2.70)	0.952	0.94 (0.34, 2.65)	0.914	0.84 (0.27, 2.66)	0.771^f^	
P for trend		0.906		0.936		0.864		

*SUA, serum uric acid; HUA, hyperuricemia; Ref, reference; HR, hazard ratio; CI, confidence interval; SD, standard deviation.*

*Model I adjusted for age, SBP, DBP, and HR.*

*Model II: ^a^Adjusted for age, SBP, DBP, HR, BMI, WHR, smoking and drinking status, Hcy, TG, HDL-C, LDL-C, eGFR, total bilirubin, AST, ALT, and antiplatelet agents. ^b^Adjusted for age, SBP, DBP, HR, BMI, WHR, smoking and drinking status, Hcy, TG, HDL-C, LDL-C, eGFR, and ALT. ^c^Adjusted for age, SBP, DBP, BMI, WHR, Hcy, HDL-C, LDL-C, and eGFR. ^d^Adjusted for age, SBP, DBP, HR, BMI, WHR, smoking and drinking status, Hcy, TG, HDL-C, LDL-C, eGFR, total bilirubin, AST, ALT, DM, antihypertensive agents, lipid-lowering agents, and antiplatelet agents. ^e^Adjusted for age, SBP, DBP, HR, BMI, Hcy, LDL-C, ALT, eGFR, and antiplatelet agents. ^f^Adjusted for age, SBP, DBP, HR, BMI, WHR, smoking and drinking status, Hcy, TG, HDL-C, LDL-C, eGFR, total bilirubin, AST, ALT, DM, and antiplatelet agents. ^g^Adjusted for age, SBP, DBP, HR, BMI, WHR, smoking and drinking status, Hcy, TG, HDL-C, LDL-C, eGFR, total bilirubin, ALT, DM, antiplatelet agents and the interaction terms for following variables: age, SBP, DBP, HR, BMI, WHR, smoking and drinking status, Hcy, TG, HDL-C, LDL-C, eGFR, total bilirubin, ALT, DM, antiplatelet agents. ^h^Adjusted for age, SBP, DBP, HR, BMI, smoking status, Hcy, TG, eGFR, ALT, antiplatelet agents and the interaction terms for following variables: age, SBP, DBP, HR, BMI, smoking status, Hcy, TG, eGFR, ALT, and antiplatelet agents. ^i^Adjusted for age, SBP, DBP, HR, BMI, WHR, smoking and drinking status, Hcy, TG, HDL-C, LDL-C, eGFR, total bilirubin, ALT, AST, antiplatelet agents and the interaction terms for following variables: age, SBP, DBP, HR, BMI, WHR, smoking and drinking status, Hcy, TG, HDL-C, LDL-C, eGFR, total bilirubin, ALT, AST, antiplatelet agents.*

**TABLE 6:** Hazard ratios of serum uric acid level categories for total first stroke events by mean arterial pressure tertiles in different models.

Variables	Event, n (%)	Crude model		Model I		Model II		*P*-value for interaction
		HR (95%CI)	*P*-value	HR (95%CI)	*P*-value	HR (95%CI)	*P*-value	
*MAP T1 [66.00, 104.65]*								
**SUA**								
Per *SD* μmol/L increase	26 (0.66%)	1.36 (0.96, 1.93)	0.084	1.27 (0.87, 1.85)	0.219	1.16 (0.75, 1.79)	0.508^a^	
**HUA**								
No	9 (0.48%)	Ref		Ref		Ref		
Yes	17 (0.83%)	1.75 (0.78, 3.92)	0.178	1.66 (0.74, 3.75)	0.220	1.42 (0.59, 3.43)	0.440^b^	
**Quartiles of SUA**								
Q1 [118.00, 325.00]	5 (0.52%)	Ref		Ref		Ref		
Q2 [326.00, 393.30]	5 (0.51%)	0.99 (0.28, 3.42)	0.983	0.89 (0.25, 3.13)	0.860	0.82 (0.23, 2.94)	0.764^c^	
Q3 [394.00, 472.00]	6 (0.62%)	1.20 (0.36, 3.93)	0.769	0.97 (0.29, 3.30)	0.963	0.83 (0.24, 2.94)	0.776^c^	
Q4 [473.00, 1029.00]	10 (0.98%)	1.91 (0.65, 5.59)	0.241	1.50 (0.48, 4.66)	0.486	1.12 (0.31, 3.98)	0.864^c^	
P for trend		0.172		0.373		0.762		
*MAP T2 [104.55, 113.22]*								
**SUA**								
Per *SD* μmol/L increase	27 (0.68%)	0.91 (0.62, 1.35)	0.637	0.87 (0.55, 1.35)	0.527	0.75 (0.45, 1.25)	0.263^d^	
**HUA**								
No	12 (0.64%)	Ref		Ref		Ref		
Yes	15 (0.72%)	1.12 (0.52, 2.39)	0.774	1.13 (0.52, 2.44)	0.755	1.07 (0.46, 2.52)	0.871^e^	
**Quartiles of SUA**								
Q1 [38.00, 325.80]	8 (0.82%)	Ref		Ref		Ref		
Q2 [326.00, 393.00]	6 (0.61%)	0.74 (0.26, 2.15)	0.584	0.66 (0.22, 1.94)	0.446	0.65 (0.22, 1.93)	0.438^f^	
Q3 [394.00, 472.00]	8 (0.79%)	0.97 (0.36, 2.60)	0.955	0.81 (0.28, 2.30)	0.690	0.79 (0.27, 2.27)	0.658^f^	
Q4 [473.00, 1056.00]	5 (0.51%)	0.63 (0.20, 1.92)	0.412	0.53 (0.16, 1.76)	0.302	0.51 (0.15, 1.74)	0.284^f^	
P for trend		0.508		0.372		0.349		
*MAP T3 [113.22, 170.89]*								
**SUA**								
Per *SD* μmol/L increase	46 (1.17%)	1.09 (0.82, 1.44)	0.567	1.02 (0.74, 1.41)	0.885	1.01 (0.69, 1.47)	0.960^g^	0.415^j^
**HUA**								
No	19 (1.00%)	Ref		Ref		Ref		0.845^k^
Yes	27 (1.32%)	1.33 (0.73, 2.39)	0.349	1.27 (0.70, 2.31)	0.432	1.30 (0.68, 2.50)	0.427^h^	
**Quartiles of SUA**								
Q1 [127.00, 325.00]	9 (0.89%)	Ref		Ref		Ref		0.826^l^
Q2 [326.00, 393.00]	12 (1.24%)	1.40 (0.59, 3.33)	0.449	1.20 (0.49, 2.89)	0.692	1.15 (0.47, 2.84)	0.758^i^	
Q3 [394.00, 472.00]	15 (1.52%)	1.72 (0.75, 3.95)	0.200	1.41 (0.59, 3.38)	0.438	1.26 (0.50, 3.18)	0.619^i^	
Q4 [473.00, 915.00]	10 (1.02%)	1.15 (0.46, 2.84)	0.765	0.93 (0.35, 2.45)	0.876	0.85 (0.29, 2.51)	0.770^i^	
P for trend		0.75		0.850		0.721		

*MAP, mean arterial pressure; SUA, serum uric acid; HUA, hyperuricemia; Ref, reference; HR, hazard ratio; CI, confidence interval; SD, standard deviation.*

*Model I adjusted for age, sex, and HR.*

*Model II: ^a^Adjusted for age, gender, HR, BMI, drinking status, Hcy, TG, LDL-C, eGFR, ALT, and antiplatelet agents. ^b^Adjusted for age, sex, HR, BMI, drinking status, Hcy, TG, LDL-C, eGFR, and antiplatelet agents. ^c^Adjusted for age, sex, HR, BMI, WHR, smoking and drinking status, Hcy, TG, HDL-C, LDL-C, eGFR, total bilirubin, ALT, DM, antihypertensive agents, lipid-lowering agents, and antiplatelet agents. ^d^Adjusted for age, sex, HR, BMI, smoking and drinking status, Hcy, TG, HDL-C, eGFR, AST, and ALT. ^e^Adjusted for age, sex, HR, BMI, smoking and drinking status, Hcy, TG, HDL-C, LDL-C, eGFR, total bilirubin, ALT, DM, antihypertensive agents, and antiplatelet agents. ^f^Adjusted for age, sex, HR, BMI and antihypertensive agents. ^g^Adjusted for age, sex, HR, BMI, WHR, smoking and drinking status, Hcy, TG, HDL-C, LDL-C, eGFR, AST, ALT, and DM. ^h^Adjusted for age, sex, BMI, WHR, smoking status, Hcy, TG, HDL-C, LDL-C, eGFR, AST and ALT. ^i^Adjusted for age, sex, BMI, WHR, smoking and drinking status, Hcy, TG, HDL-C, LDL-C, eGFR, AST, and ALT. ^j^Adjusted for age, HR, BMI, WHR, smoking and drinking status, Hcy, TG, HDL-C, LDL-C, eGFR, total bilirubin, ALT, DM, antiplatelet agents and the interaction terms for following variables: age, WHR, smoking and drinking status, Hcy, TG, LDL-C, eGFR, total bilirubin, and ALT. ^k^Adjusted for age, sex, HR, BMI, smoking status, Hcy, TG, eGFR, ALT, antiplatelet agents and the interaction terms for following variables: age, sex, HR, BMI, smoking status, Hcy, TG, eGFR, ALT, antiplatelet agents. ^l^Adjusted for age, sex, HR, BMI, WHR, smoking and drinking status, Hcy, TG, HDL-C, LDL-C, eGFR, total bilirubin, ALT, AST, antiplatelet agents and the interaction terms for following variables: age, sex, HR, BMI, WHR, smoking and drinking status, Hcy, TG, HDL-C, LDL-C, eGFR, total bilirubin, ALT, AST, antiplatelet agents.*

**TABLE 7:** Hazard ratios of serum uric acid level categories for total first stroke events by antihypertensive drugs usage in different models.

Variables	Event, n (%)	Crude model		Model I		Model II		*P*-value for interaction
		HR (95%CI)	*P*-value	HR (95%CI)	*P*-value	HR (95%CI)	*P*-value	
*Non-use of antihypertensive drugs*								
**SUA**								
Per *SD* μmol/L increase	38 (0.85%)	0.87 (0.62, 1.22)	0.424	0.76 (0.52, 1.12)	0.168	0.73 (0.46, 1.16)	0.179^a^	
**HUA**								
No	21 (0.94%)	Ref		Ref		Ref		
Yes	17 (0.76%)	0.81 (0.42, 1.53)	0.511	0.80 (0.41, 1.53)	0.497	0.80 (0.39, 1.63)	0.533^b^	
**Quartiles of SUA**								
Q1 [93.00, 325.00]	11 (0.92%)	Ref		Ref		Ref		
Q2 [326.00, 393.30]	10 (0.90%)	0.98 (0.42, 2.32)	0.967	0.84 (0.35, 2.03)	0.694	0.79 (0.31, 1.99)	0.619^c^	
Q3 [394.00, 472.00]	10 (0.91%)	1.00 (0.42, 2.35)	0.992	0.78 (0.31, 1.95)	0.599	0.74 (0.28, 1.97)	0.545^c^	
Q4 [473.00, 913.00]	7 (0.66%)	0.71 (0.28, 1.85)	0.488	0.53 (0.19, 1.49)	0.228	0.49 (0.15, 1.58)	0.230^c^	
P for trend		0.510		0.229		0.237		
*Use of antihypertensive drugs*								
**SUA**								
Per *SD* μmol/L increase	61 (0.83%)	1.26 (1.00, 1.58)	0.055	1.22 (0.94, 1.58)	0.128	1.11 (0.83, 1.48)	0.483^d^	0.070^g^
**HUA**								
No	19 (0.56%)	Ref		Ref		Ref		0.094^h^
Yes	42 (1.06%)	1.93 (1.12, 3.32)	0.018	1.82 (1.05, 3.15)	0.032	1.67 (0.94, 2.99)	0.083^e^	
**Quartiles of SUA**								
Q1 [38.00, 325.80]	11 (0.62%)	Ref		Ref		Ref		0.813^i^
Q2 [326.00, 393.30]	13 (0.71%)	1.14 (0.51, 2.56)	0.747	1.02 (0.45, 2.31)	0.957	0.95 (0.41, 2.17)	0.898^f^	
Q3 [394.00, 472.00]	19 (1.02%)	1.63 (0.77, 3.44)	0.198	1.36 (0.62, 2.96)	0.439	1.14 (0.51, 2.55)	0.758^f^	
Q4 [473.00, 1056.00]	18 (0.94%)	1.51 (0.71, 3.21)	0.282	1.27 (0.57, 2.82)	0.560	0.96 (0.40, 2.30)	0.919^f^	
P for trend		0.217		0.483		0.952		

*SUA, serum uric acid; HUA, hyperuricemia; Ref, reference; HR, hazard ratio; CI, confidence interval; SD, standard deviation.*

*Model I adjusted for age, sex, SBP, DBP, and HR.*

*Model II: ^a^Adjusted for age, sex, SBP, DBP, BMI, WHR, smoking, and drinking status, Hcy, TG, HDL-C, LDL-C, eGFR, AST, and ALT. ^b^Adjusted for age, sex, SBP, DBP, BMI, WHR, drinking status, Hcy, TG, HDL-C, LDL-C, eGFR, AST, and ALT. ^c^Adjusted for age, sex, SBP, DBP, HR, BMI, WHR, smoking and drinking status, Hcy, TG, HDL-C, LDL-C, eGFR, total bilirubin, AST, ALT, DM, lipid-lowering agents, and antiplatelet agents. ^d^Adjusted for age, sex, SBP, HR, BMI, smoking status, Hcy, LDL-C, eGFR, ALT, and antiplatelet agents. ^e^Adjusted for age, sex, SBP, HR, BMI, smoking status, Hcy, eGFR, and antiplatelet agents. ^f^Adjusted for age, sex, SBP, DBP, HR, BMI, smoking and drinking status, Hcy, HDL-C, LDL-C, eGFR, AST, ALT, total bilirubin, and antiplatelet agents. ^g^Adjusted for age, sex, SBP, DBP, HR, BMI, WHR, smoking, and drinking status, Hcy, TG, HDL-C, LDL-C, eGFR, total bilirubin, ALT, DM, antiplatelet agents and the interaction terms for following variables: sex, smoking, and eGFR. ^h^Adjusted for age, sex, SBP, DBP, HR, BMI, smoking status, Hcy, TG, eGFR, ALT, antiplatelet agents and the interaction terms for following variables: age, sex, SBP, DBP, HR, BMI, smoking status, TG, eGFR, ALT, antiplatelet agents. ^i^Adjusted for age, sex, SBP, DBP, HR, BMI, WHR, smoking and drinking status, Hcy, TG, HDL-C, LDL-C, eGFR, total bilirubin, ALT, AST, antiplatelet agents and the interaction terms for following variables: age, sex, SBP, DBP, HR, BMI, WHR, smoking and drinking status, TG, HDL-C, LDL-C, eGFR, total bilirubin, ALT, AST, antiplatelet agents.*

## Discussion

We found that increased SUA levels were not significantly associated with the first stroke event in Chinese adults with hypertension. However, age played an interactive role in the relationship between HUA and the first stroke event. In the population, less than 60 years old subjects with HUA had a significantly higher risk of the first stroke than the population without HUA. In subjects older than 60 years, we did not find a significant relationship between HUA and first stroke.

Uric acid is an end enzymatic product of purine metabolism in humans, which can scavenge hydroxyl radicals or hydrogen peroxide and prevent lipid peroxidation (Becker, 1993; Sautin and Johnson, 2008; Sinha et al., 2009). The adaptive advantages of uric acid elevation as a result of a mutation in the uracase gene are at least partially attributable to uric acid antioxidant properties. It has been also suggested that the loss of the uricase gene and subsequent uric acid elevation may have occurred to compensate for reduced plasma antioxidant activity after the loss of ascorbate synthesis (Ames et al., 1981). Higher SUA levels have been related to better reaction ability to control BP at lower salt intake (Watanabe et al., 2002). Demonstration of lower uric acid levels in several neurodegenerative diseases, such as multiple sclerosis, Parkinson’s disease, and Alzheimer’s disease, has fueled the hypothesis that uric acid may be neuroprotective (Toncev et al., 2002; Irizarry et al., 2009; Kuo et al., 2010). Experimental evidence suggests that uric acid may serve as an immunity stimulator. Studies in mice showed that uric acid is released from injured somatic cells and functions as an innate immunity enhancer by stimulating the maturation of dendritic cells and antigen-presenting cells to endogenous antigens (Shi et al., 2003). Monosodium uric acid crystals activate the inflammasome participating in innate immunity and initiation of inflammation (Martinon et al., 2006).

Most epidemiological studies but not all of them suggested the existence of an association between elevated SUA levels and cardiovascular diseases (CVDs), such as stroke (Lehto et al., 1998; Chien et al., 2005; Bos et al., 2006; Gerber et al., 2006; Hozawa et al., 2006; Kim et al., 2009; Chao et al., 2014; Li et al., 2014; Shi et al., 2017; Tu et al., 2019; Li J. et al., 2020; Zhang et al., 2020), CHD, arterial hypertension, and an increased risk for mortality due to CVDs in general population and subjects with confirmed CHD (Ndrepepa, 2018b; Khalil et al., 2020; Tatsumi et al., 2020). In the Saku study, HUA was found to be independently associated with the development of hypertension independent of alcohol drinking status (Ndrepepa, 2018b). Experimental and clinical studies have evidenced several mechanisms through which elevated UA level exerts deleterious effects on cardiovascular health including increased oxidative stress, reduced availability of nitric oxide and endothelial dysfunction, promotion of local and systemic inflammation, vasoconstriction and proliferation of vascular smooth muscle cells, insulin resistance, and metabolic dysregulation (Tatsumi et al., 2020).

Furthermore, some previous studies indicated that the influence of SUA on stroke was due to the secondary association of SUA with other established etiological risk factors, including hypertension, arterial stiffness, obesity, and hyperinsulinemia (Lehto et al., 1998; Hozawa et al., 2006; Ishizaka et al., 2007; Liang et al., 2009; Shi et al., 2017; Chaudhary et al., 2020). Multiple lines of evidence from epidemiological (Perlstein et al., 2006; Gaffo et al., 2013), animal (Kang et al., 2005; Corry et al., 2008) studies, and clinical trials (Feig et al., 2008) suggested that SUA might increase BP. The mechanism where elevated SUA levels induced hypertension remained elusive, involving crystal pathway, activation of the intrarenal renin-angiotensin system, reducing endothelial nitric oxide synthase phosphorylation, the elevation of aldose reductase, mitochondrial dysfunction, and superoxide generation (Lanaspa et al., 2020). Some studies indicated that hypertension might mediate the effect of HUA on stroke risk (Hozawa et al., 2006; Shi et al., 2017; Chaudhary et al., 2020). In the Atherosclerosis Risk in Communities (ARIC) study, over 13,000 participants were followed for incident stroke over 12.6 years, HUA was no longer independently associated with ischemic stroke after adjustment for diuretic-treated hypertension (Hozawa et al., 2006). In the REGARDS study, apparent treatment-resistant hypertension and separately count of antihypertensive medication classes significantly reduced the effect of HUA on ischemic stroke (Chaudhary et al., 2020). The evidence available also suggests an association between elevated UA and traditional cardiovascular risk factors, metabolic syndrome, insulin resistance, obesity, non-alcoholic fatty liver disease, and CKD (Tatsumi et al., 2020). Thus, some scholars hypothesized that uric acid may be pathogenic and participate in the pathophysiology of CVDs by serving as a bridging mechanism mediating or potentiating the deleterious effects of cardiovascular risk factors on vascular tissue and myocardium (Ndrepepa, 2018a; Tatsumi et al., 2020).

The role of HUA as an independent risk factor for stroke in Chinese hypertensive patients was still controversial (Chien et al., 2005; Bos et al., 2006; Shi et al., 2017; Zhang et al., 2020). Baseline or time-dependent elevated SUA had a significant risk for stroke only in hypertension and metabolic syndrome subgroup across populations with relatively low CHD but high stroke risk in Taiwan (Chien et al., 2005). The Rotterdam study indicated that elevated SUA was a strong risk factor for stroke and this positive relationship was stronger in the population without hypertension than in those with hypertension (Bos et al., 2006). Increased SUA levels were positively associated with ischemic stroke and HUA has a good predictive value for ischemic stroke among hypertensive participants in the Chinese community (Zhang et al., 2020). Although elevated SUA was not significantly associated with the risk of the first stroke, there was a statistically significant decreased risk of hemorrhagic stroke for the second quartile of SUA levels compared to the first quartile in a Chinese population of hypertensive patients (Shi et al., 2017). Considering the rate differences of HUA in different sex and population attributable fractions across hypertensive patients, some previous studies suggested that women had a high risk of SUA on stroke events (Chien et al., 2005; Li J. et al., 2020). However, no studies have been reported that age could modify the association between increased SUA levels and stroke events. To our knowledge, this was the first discovery that age played an interactive role between HUA with the first stroke event. In our analysis, subjects aged less than 60 years old had a higher prevalence of general or central obesity and dyslipidemia than the aging group (Supplementary Table 2), which might explain the interactive role of aging between HUA with the first stroke event. Therefore, targeted stroke prevention should include healthy lifestyle improvement and treatment of metabolic syndrome in an individualized and comprehensive way, especially younger Chinese adults with hypertension (Pandian et al., 2018).

Furthermore, current evidence showed that SUA levels increased rapidly after acute ischemic stroke (AIS). Nevertheless, the relationship between SUA levels and AIS outcome remained debatable (Maloberti et al., 2020). Animal models of AIS showed that SUA might be neuroprotective (Yu et al., 1998). In humans, HUA might be an independent predictor of better outcome after AIS (Chamorro et al., 2002). However, the primer registro mexicano de isquemia cerebral (PREMIER) study revealed that a low SUA concentration was modestly associated with a very good short-term outcome (12-month follow-up) (Chiquete et al., 2013). Wang et al. (2019) found that HUA independently predicted the poor in-hospital outcome of AIS in diabetic patients, especially in patients aged less than 75 years old.

Moreover, no evidence indicated that lowering SUA levels with drug treatment had a beneficial effect on stroke outcome. Although meta-analyses on randomized controlled trials (RCTs) suggest cardiovascular benefits with allopurinol, few high-quality RCTs have examined the allopurinol effect of urate-lowering therapy among patients with HUA or gout (Kang and Kim, 2019). Larsen et al. (2016) showed that lowering of urate by allopurinol improved cardiovascular outcomes including stroke among patients with HUA. Yen et al. (2020) found that uricosuric agents and xanthine oxidase inhibitors significantly mitigated the risks of hospitalized stroke and all-cause mortality in patients with gout. We eagerly await results from ongoing large-scale RCTs of urate-lowering therapy on various clinical cardiovascular outcomes.

Considering that the influence of SUA on the risk of stroke events might due to the secondary association of SUA with other traditional risk factors, such as hypertension and obesity (Lehto et al., 1998; Hozawa et al., 2006; Ishizaka et al., 2007; Liang et al., 2009; Shi et al., 2017; Chaudhary et al., 2020). We did subgroup analyses in the mean arterial pressure tertiles, SBP tertiles, DBP tertiles, antihypertensive drugs usage, body mass index tertiles, and central obesity (Tables 6, 7, Supplementary Figure 3, and Supplementary Tables 5–8). Nevertheless, these above covariates could not modify the relationship between increased SUA levels and the first stroke event.

Several limitations of our study should be addressed in interpreting the results. First, we did not test the excretion or metabolite rate of SUA, which could affect the SUA levels. Second, the levels of SUA were measured only once at baseline, and multiple determinations might be necessary to evaluate the kinetics of SUA levels. In addition, our follow-up period was not long, and fewer stroke events occurred. The relatively low incidence of stroke events reduced the strength of this finding.

## Conclusion

To sum up, no significant evidence in the present study indicated that increased SUA levels were associated with the risk of the first stroke in Chinese adults with hypertension. Nonetheless, age played an interactive role in the relationship between HUA and the first stroke event. In the population less than 60 years old, subjects with HUA had a significantly higher risk of the first stroke than the population without HUA. In subjects older than 60 years, we did not find a significant relationship between HUA and first stroke. Definitive proof of causality and the mechanism between SUA levels and stroke outcomes requires an appropriately designed therapeutic controlled trial.

## Data Availability Statement

The original contributions presented in the study are included in the article/Supplementary Material, further inquiries can be directed to the corresponding author/s.

## Ethics Statement

The studies involving human participants were reviewed and approved by the Ethics Committee of the Institute of Biomedicine, Anhui Medical University. The patients/participants provided their written informed consent to participate in this study.

## Author Contributions

FH: conception and design, data acquisition and analysis, interpretation, drafting, and final approval. LH: conception and design, data acquisition and analysis, interpretation, critical revision, and final approval. RY and FYH: data acquisition and final approval. WZ and TW: design and final approval. LZ and XH: analysis and interpretation and final approval. HB and XC: conception and design and critical revision and final approval. All authors contributed to the article and approved the submitted version.

## Conflict of Interest

The authors declare that the research was conducted in the absence of any commercial or financial relationships that could be construed as a potential conflict of interest.

## Publisher’s Note

All claims expressed in this article are solely those of the authors and do not necessarily represent those of their affiliated organizations, or those of the publisher, the editors and the reviewers. Any product that may be evaluated in this article, or claim that may be made by its manufacturer, is not guaranteed or endorsed by the publisher.

## Footnotes

1
http://www.R-project.org

2
www.empowerstats.com

## Supplementary Material

Supplementary Figure 1 The smooth curve of correlation between SUA levels and stroke occur probability. Smooth curve adjusted for age, sex, SBP, DBP, HR, BMI, WHR, smoking and drinking status, Hcy, TG, HDL-C, LDL-C, eGFR, ALT, total bilirubin, DM, and antiplatelet agents. This analysis was conducted using both logarithmic transformed and untransformed data. Log (relative risk, RR) can be converted to a relative risk by taking antilog. For example, a log RR of 0 implies the relative risk of 1 (no impact on the probability of death), whereas a log RR of 1 implies the relative risk of 2.71 (i.e., 2.71-fold increase in the probability of death). The piece-wise regression suggested that the relationship between SUA levels and the risk of the first stroke was linear (*P*-value of Log-likelihood ratio test = 0.31). eGFR, estimated glomerular filtration rate; SBP, systolic blood pressure; DBP, diastolic blood pressure; HR, heart rate, BMI, body mass index; Hcy, homocysteine; TG, total triglyceride; HDL-C, high-density lipoprotein cholesterol; LDL-C, low-density lipoprotein cholesterol; AST, aspartate aminotransferase; ALT, alanine aminotransferase.

Supplementary Figure 2 The cumulative total first stroke risks in the study population were grouped by sex.**(A)** adjusted for age, SBP, DBP, HR, BMI, WHR, smoking and drinking status, Hcy, TG, HDL-C, LDL-C, eGFR, and ALT.**(B)** Adjusted for age, SBP, DBP, HR, BMI, Hcy, LDL-C, ALT, eGFR, and antiplatelet agents. eGFR, estimated glomerular filtration rate; SBP, systolic blood pressure; DBP, diastolic blood pressure; HR, heart rate, BMI, body mass index; Hcy, homocysteine; TG, total triglyceride; HDL-C, high-density lipoprotein cholesterol; LDL-C, low-density lipoprotein cholesterol; AST, aspartate aminotransferase; ALT, alanine aminotransferase.

Supplementary Figure 3 The cumulative total first stroke risks in the study population grouped by mean arterial pressure. **(A)** Adjusted for age, sex, HR, BMI, drinking status, Hcy, TG, LDL-C, eGFR, and antiplatelet agents. **(B)** Adjusted for age, sex, HR, BMI, smoking and drinking status, Hcy, TG, HDL-C, LDL-C, eGFR, total bilirubin, ALT, DM, antihypertensive agents, and antiplatelet agents. **(C)** Adjusted for age, sex, BMI, WHR, smoking status, Hcy, TG, HDL-C, LDL-C, eGFR, AST, and ALT. MAP, mean arterial pressure; eGFR, estimated glomerular filtration rate; HR, heart rate, BMI, body mass index; Hcy, homocysteine; TG, total triglyceride; HDL-C, high-density lipoprotein cholesterol; LDL-C, low-density lipoprotein cholesterol; AST, aspartate aminotransferase; ALT, alanine aminotransferase.

Supplementary Figure 4 The smooth curve of correlation between age and serum uric acid levels.
