# Supplementary figures and images for: Prospective Study of Serum Uric Acid Levels and First Stroke Events in Chinese Adults With Hypertension
Source: Front Physiol. 2021 Dec 23;12:807420. doi: 10.3389/fphys.2021.807420 (PMC8733595; doi:10.3389/fphys.2021.807420)

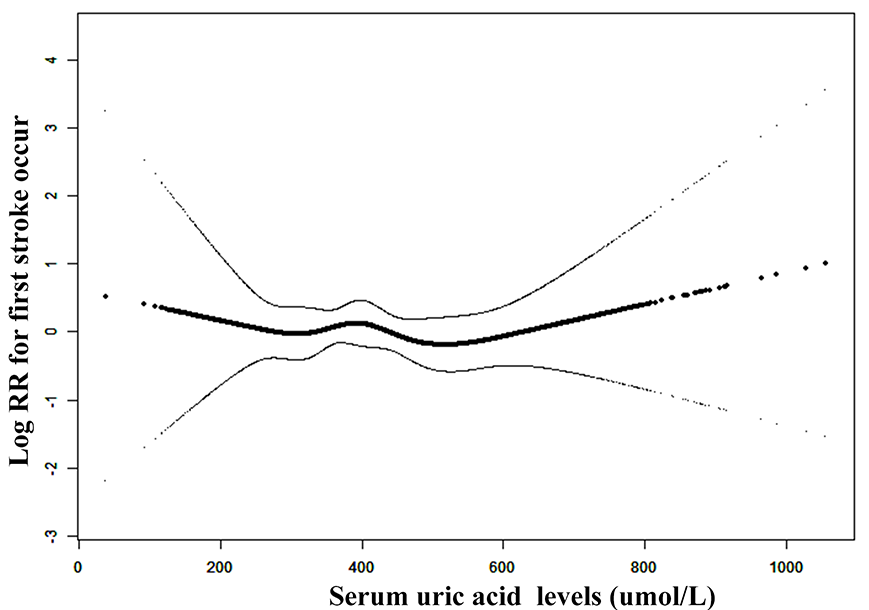

Supplement: Supplementary Figure 1 — The smooth curve of correlation between SUA levels and stroke occur probability. Smooth curve adjusted for age, sex, SBP, DBP, HR, BMI, WHR, smoking and drinking status, Hcy, TG, HDL-C, LDL-C, eGFR, ALT, total bilirubin, DM, and antiplatelet agents. This analysis was conducted using both logarithmic transformed and untransformed data. Log (relative risk, RR) can be converted to a relative risk by taking antilog. For example, a log RR of 0 implies the relative risk of 1 (no impact on the probability of death), whereas a log RR of 1 implies the relative risk of 2.71 (i.e., 2.71-fold increase in the probability of death). The piece-wise regression suggested that the relationship between SUA levels and the risk of the first stroke was linear (P-value of Log-likelihood ratio test = 0.31). eGFR, estimated glomerular filtration rate; SBP, systolic blood pressure; DBP, diastolic blood pressure; HR, heart rate, BMI, body mass index; Hcy, homocysteine; TG, total triglyceride; HDL-C, high-density lipoprotein cholesterol; LDL-C, low-density lipoprotein cholesterol; AST, aspartate aminotransferase; ALT, alanine aminotransferase. [file Image_1.TIF]

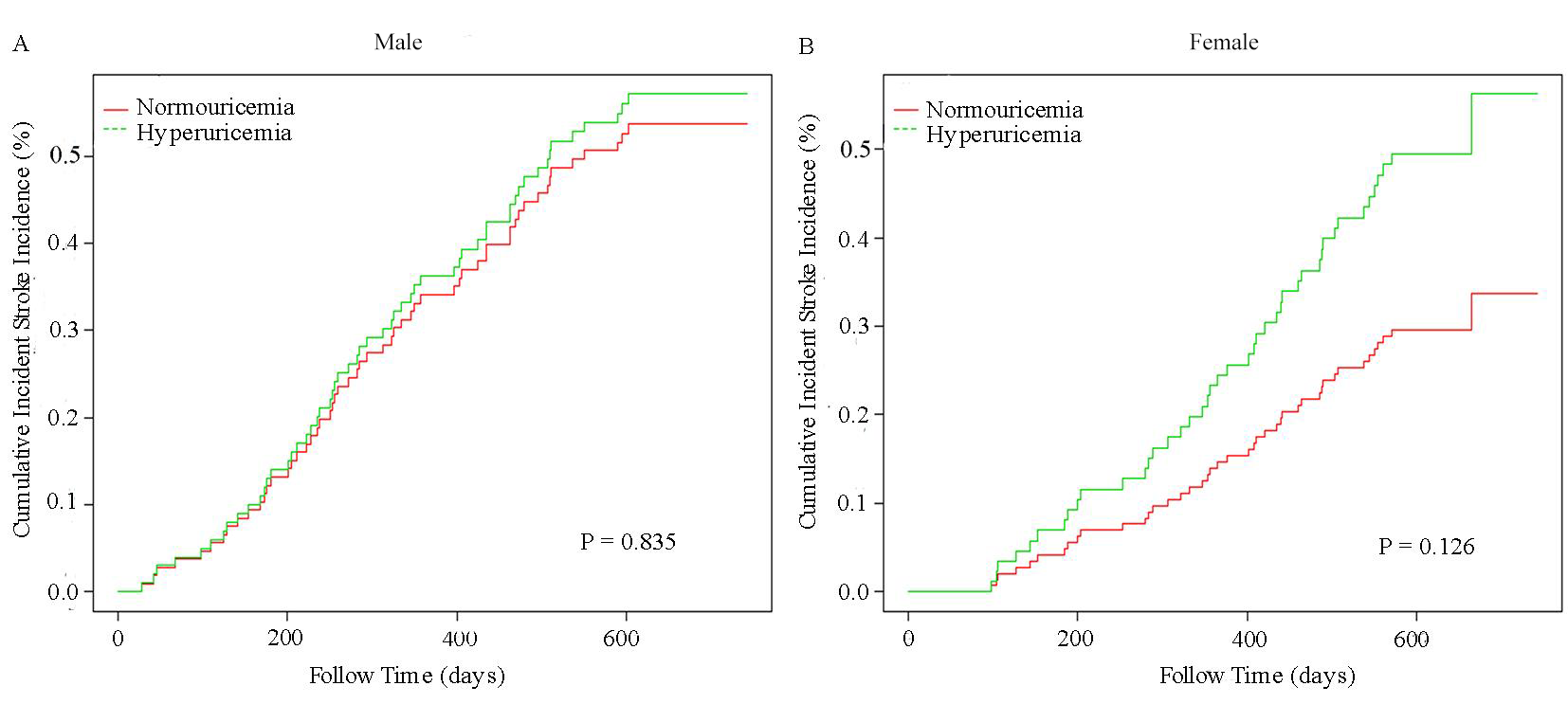

Supplement: Supplementary Figure 2 — The cumulative total first stroke risks in the study population were grouped by sex.(A) adjusted for age, SBP, DBP, HR, BMI, WHR, smoking and drinking status, Hcy, TG, HDL-C, LDL-C, eGFR, and ALT.(B) Adjusted for age, SBP, DBP, HR, BMI, Hcy, LDL-C, ALT, eGFR, and antiplatelet agents. eGFR, estimated glomerular filtration rate; SBP, systolic blood pressure; DBP, diastolic blood pressure; HR, heart rate, BMI, body mass index; Hcy, homocysteine; TG, total triglyceride; HDL-C, high-density lipoprotein cholesterol; LDL-C, low-density lipoprotein cholesterol; AST, aspartate aminotransferase; ALT, alanine aminotransferase. [file Image_2.TIF]

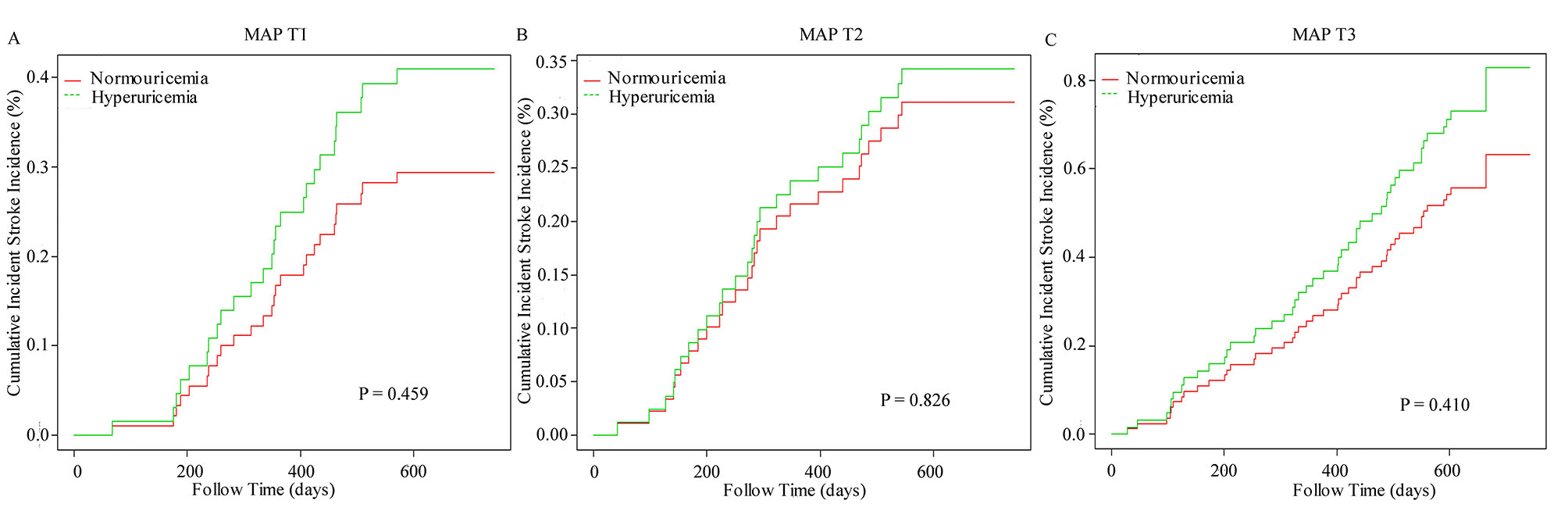

Supplement: Supplementary Figure 3 — The cumulative total first stroke risks in the study population grouped by mean arterial pressure. (A) Adjusted for age, sex, HR, BMI, drinking status, Hcy, TG, LDL-C, eGFR, and antiplatelet agents. (B) Adjusted for age, sex, HR, BMI, smoking and drinking status, Hcy, TG, HDL-C, LDL-C, eGFR, total bilirubin, ALT, DM, antihypertensive agents, and antiplatelet agents. (C) Adjusted for age, sex, BMI, WHR, smoking status, Hcy, TG, HDL-C, LDL-C, eGFR, AST, and ALT. MAP, mean arterial pressure; eGFR, estimated glomerular filtration rate; HR, heart rate, BMI, body mass index; Hcy, homocysteine; TG, total triglyceride; HDL-C, high-density lipoprotein cholesterol; LDL-C, low-density lipoprotein cholesterol; AST, aspartate aminotransferase; ALT, alanine aminotransferase. [file Image_3.JPEG]

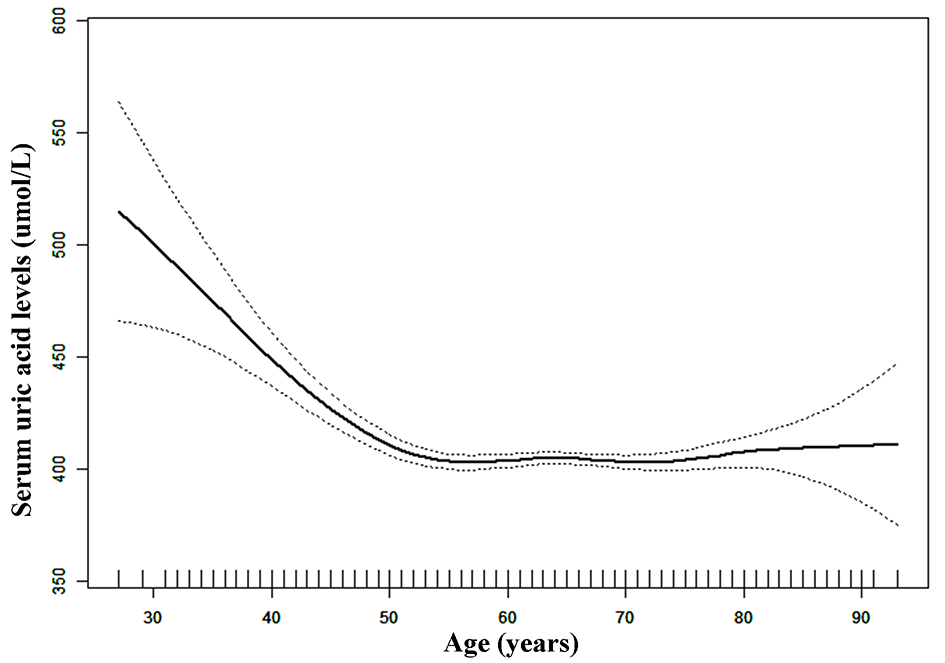

Supplement: Supplementary Figure 4 — The smooth curve of correlation between age and serum uric acid levels. [file Image_4.TIF]
