# Supplementary material for: Prospective Study of Serum Uric Acid Levels and First Stroke Events in Chinese Adults With Hypertension
Source: Front Physiol. 2021 Dec 23;12:807420. doi: 10.3389/fphys.2021.807420 (PMC8733595; doi:10.3389/fphys.2021.807420)
Supplement: Supplementary file 5 [file Table_1.docx]

**Supplemenary Table Legends**

| **Supplemenary Table 1. Associations of covariates with incident stroke.** | | | | | | | | |
| --- | --- | --- | --- | --- | --- | --- | --- | --- |
| **Covariates** | **Total stroke** | |  | **Ischemic stroke** | |  | **Hemorrhagic stroke** | |
|  | ***HR (95%CI)*** | ***P*-value** |  | ***HR (95%CI)*** | ***P*-value** |  | ***HR (95%CI)*** | ***P*-value** |
| Age (years) | 1.06 (1.04, 1.09) | <0.001 |  | 1.07 (1.04, 1.11) | <0.001 |  | 0.95 (0.90, 1.01) | 0.0893 |
| Gender, n(%) |  |  |  |  |  |  |  |  |
| Male | *Ref* |  |  | *Ref* |  |  | *Ref* |  |
| Female | 0.64 (0.43, 0.96) | 0.0301 |  | 0.75 (0.43, 1.29) | 0.298 |  | 0.31 (0.10, 0.96) | 0.042 |
| SBP (mmHg) | 1.02 (1.01, 1.04) | <0.001 |  | 1.02 (1.01, 1.04) | 0.004 |  | 1.04 (1.01, 1.06) | 0.004 |
| DBP (mmHg) | 1.03 (1.01, 1.04) | 0.006 |  | 1.01 (0.98, 1.03) | 0.695 |  | 1.12 (1.07, 1.16) | <0.001 |
| HR (times/min) | 1.01 (1.00, 1.03) | 0.014 |  | 1.00 (0.98, 1.02) | 0.826 |  | 1.03 (1.00, 1.05) | 0.018 |
| BMI (kg/m^^2^) | 0.91 (0.86, 0.97) | 0.003 |  | 0.95 (0.88, 1.03) | 0.233 |  | 0.99 (0.86, 1.15) | 0.906 |
| WHR | 0.41 (0.03, 6.46) | 0.526 |  | 1.01 (0.21, 4.84) | 0.994 |  | 0.01 (0.00, 6.45) | 0.149 |
| Smoking status, n(%) |  |  |  |  |  |  |  |  |
| Never | *Ref* |  |  | *Ref* |  |  | *Ref* |  |
| Former | 1.15 (0.63, 2.08) | 0.652 |  | 1.08 (0.47, 2.49) | 0.857 |  | 0.67 (0.08, 5.55) | 0.709 |
| Current | 1.70 (1.11, 2.62) | 0.016 |  | 1.60 (0.88, 2.92) | 0.126 |  | 3.08 (1.07, 8.89) | 0.037 |
| Drinking status, n(%) |  |  |  |  |  |  |  |  |
| Never | *Ref* |  |  | *Ref* |  |  | *Ref* |  |
| Former | 1.26 (0.70, 2.28) | 0.433 |  | 0.71 (0.25, 2.02) | 0.518 |  | 1.54 (0.42, 5.61) | 0.511 |
| Current | 1.36 (0.87, 2.15) | 0.180 |  | 1.72 (0.96, 3.11) | 0.070 |  | 0.55 (0.12, 2.53) | 0.446 |
| Hcy (μmol/L) | 1.02 (1.01, 1.03) | <0.001 |  | 1.02 (1.01, 1.04) | <0.001 |  | 1.02 (0.98, 1.05) | 0.340 |
| TC (mmol/L) | 0.96 (0.80, 1.15) | 0.639 |  | 1.21 (0.95, 1.53) | 0.125 |  | 0.65 (0.39, 1.07) | 0.093 |
| TG (mmol/L) | 0.95 (0.80, 1.13) | 0.558 |  | 1.11 (0.95, 1.31) | 0.194 |  | 0.51 (0.23, 1.13) | 0.095 |
| HDL-C (mmol/L) | 0.75 (0.47, 1.22) | 0.254 |  | 0.81 (0.42, 1.58) | 0.536 |  | 0.39 (0.10, 1.51) | 0.172 |
| LDL-C (mmol/L) | 1.00 (0.79, 1.29) | 0.969 |  | 1.37 (1.00, 1.87) | 0.050 |  | 0.48 (0.23, 0.99) | 0.048 |
| eGFR (ml/min/1.73m^2^) | 0.97 (0.96, 0.98) | <0.001 |  | 0.96 (0.95, 0.98) | <0.001 |  | 0.96 (0.93, 1.00) | 0.034 |
| Total bilirubin (mmol/L) | 1.01 (0.99, 1.03) | 0.316 |  | 0.99 (0.95, 1.03) | 0.634 |  | 1.02 (0.99, 1.04) | 0.151 |
| Direct bilirubin (mmol/L) | 1.02 (0.99, 1.05) | 0.257 |  | 0.97 (0.85, 1.11) | 0.667 |  | 1.02 (0.97, 1.08) | 0.406 |
| AST (U/L) | 1.00 (0.99, 1.02) | 0.793 |  | 1.00 (0.97, 1.02) | 0.764 |  | 0.96 (0.90, 1.03) | 0.293 |
| ALT (U/L) | 0.99 (0.97, 1.01) | 0.252 |  | 0.98 (0.95, 1.01) | 0.185 |  | 0.99 (0.96, 1.04) | 0.803 |
| DM, n(%) |  |  |  |  |  |  |  |  |
| No | *Ref* |  |  | *Ref* |  |  | *Ref* |  |
| Yes | 1.20 (0.73, 1.97) | 0.466 |  | 1.30 (0.67, 2.55) | 0.436 |  | 0.34 (0.04, 2.57) | 0.295 |
| Antihypertensive agents, n(%) |  |  |  |  |  |  |  |  |
| No | *Ref* |  |  | *Ref* |  |  | *Ref* |  |
| Yes | 0.97 (0.65, 1.46) | 0.901 |  | 1.22 (0.68, 2.18) | 0.512 |  | 0.53 (0.19, 1.46) | 0.221 |
| Lipid-lowering agents, n(%) |  |  |  |  |  |  |  |  |
| No | *Ref* |  |  | *Ref* |  |  | *Ref* |  |
| Yes | 1.27 (0.40, 4.03) | 0.685 |  | 1.66 (0.40, 6.86) | 0.484 |  | 2.90 (0.38, 22.15) | 0.304 |
| Antiplatelet agents, n(%) |  |  |  |  |  |  |  |  |
| No | *Ref* |  |  | *Ref* |  |  | *Ref* |  |
| Yes | 2.35 (0.95, 5.82) | 0.065 |  | 1.79 (0.43, 7.40) | 0.422 |  | 6.76 (1.52, 30.12) | 0.012 |
| Abbreviations: SBP, systolic blood pressure; DBP, diastolic blood pressure; HR, heart rate; BMI, body mass index; WHR, waist hip rate; Hcy, homocysteine; TC, total cholesterol; TG, total triglyceride; HDL-C, high-density lipoprotein cholesterol; LDL-C, low-density lipoprotein cholesterol; eGFR, estimated glomerular filtration rate; AST, aspartate aminotransferase; ALT, alanine aminotransferase; CHD, coronary heart disease; DM, diabetes mellitus; *Ref*, reference; *HR*, hazard ratio; *CI*, confidence interval. | | | | | | | | |

| **Supplemenary Table 2. Clinical characteristics of the study population grouped by sex or age.** | | | | | | | |
| --- | --- | --- | --- | --- | --- | --- | --- |
| **Characteristics** | **Age** | | |  | **Sex** | | |
|  | **<60 years** | **≥60 years** | ***P-value*** |  | **Male** | **Female** | ***P-value*** |
| Number of subjects (n) | 3927 | 7914 |  |  | 5408 | 6433 |  |
| Age (years) | 52.57 ± 4.96 | 68.11 ± 5.73 | <0.001 |  | 62.85 ± 9.63 | 63.04 ± 8.72 | 0.914 |
| Male, n(%) | 1798 (45.79%) | 3610 (45.62%) | 0.861 |  |  |  |  |
| SBP (mmHg) | 146.33 ± 16.42 | 149.61 ± 17.72 | <0.001 |  | 146.29 ± 17.27 | 150.40 ± 17.23 | <0.001 |
| DBP (mmHg) | 94.38 ± 9.49 | 87.00 ± 10.13 | <0.001 |  | 90.85 ± 10.71 | 88.27 ± 10.19 | <0.001 |
| MAP (mmHg) | 111.70 ± 10.60 | 107.87 ± 11.07 | <0.001 |  | 109.33 ± 11.27 | 108.98 ± 10.89 | 0.045 |
| HR (times/min) | 77.52 ± 12.44 | 76.08 ± 14.46 | <0.001 |  | 74.27 ± 13.33 | 78.48 ± 13.97 | <0.001 |
| BMI (kg/m^^2^) | 25.02 ± 3.41 | 23.07 ± 3.42 | <0.001 |  | 23.53 ± 3.48 | 23.87 ± 3.59 | <0.001 |
| BMI group (kg/m^^2^) |  |  | <0.001 |  |  |  | <0.001 |
| Control (<24) | 1557 (39.67%) | 4964 (62.76%) |  |  | 3046 (56.33%) | 3475 (54.06%) |  |
| Overweight (≥24, <28) | 1702 (43.36%) | 2339 (29.57%) |  |  | 1827 (33.79%) | 2214 (34.44%) |  |
| General obesity (≥28) | 666 (16.97%) | 607 (7.67%) |  |  | 534 (9.88%) | 739 (11.50%) |  |
| WHR | 0.92 ± 0.27 | 0.91 ± 0.07 | <0.001 |  | 0.92 ± 0.24 | 0.91 ± 0.08 | <0.001 |
| Central obesity, n(%) | 2955 (75.25%) | 5467 (69.08%) | <0.001 |  | 3271 (60.48%) | 5151 (80.07%) | <0.001 |
| Smoking status, n(%) |  |  | <0.001 |  |  |  | <0.001 |
| Never | 2622 (66.79%) | 4413 (55.77%) |  |  | 1173 (21.69%) | 5862 (91.14%) |  |
| Former | 419 (10.67%) | 1335 (16.87%) |  |  | 1530 (28.30%) | 224 (3.48%) |  |
| Current | 885 (22.54%) | 2165 (27.36%) |  |  | 2704 (50.01%) | 346 (5.38%) |  |
| Drinking status, n(%) |  |  | <0.001 |  |  |  | <0.001 |
| Never | 2585 (65.84%) | 5026 (63.52%) |  |  | 2010 (37.17%) | 5601 (87.08%) |  |
| Former | 414 (10.55%) | 1067 (13.48%) |  |  | 1004 (18.57%) | 477 (7.42%) |  |
| Current | 927 (23.61%) | 1820 (23.00%) |  |  | 2393 (44.26%) | 354 (5.50%) |  |
| Hcy (μmol/L) | 13.37 (11.57-16.38) | 15.03 (12.69-18.59) | <0.001 |  | 15.83 (13.21-19.98) | 13.46 (11.68-16.27) | <0.001 |
| FBG (mmol/L) | 6.23 ± 1.69 | 6.15 ± 1.56 | 0.015 |  | 6.07 ± 1.49 | 6.26 ± 1.69 | <0.001 |
| TC (mmol/L) | 5.20 ± 1.10 | 5.18 ± 1.09 | 0.381 |  | 4.98 ± 1.03 | 5.37 ± 1.11 | <0.001 |
| TG (mmol/L) | 1.70 (1.20-2.54) | 1.38 (0.99-1.99) | <0.001 |  | 1.32 (0.93-2.02) | 1.59 (1.17-2.27) | <0.001 |
| HDL-C (mmol/L) | 1.51 ± 0.41 | 1.62 ± 0.43 | <0.001 |  | 1.55 ± 0.44 | 1.61 ± 0.42 | <0.001 |
| LDL-C (mmol/L) | 3.07 ± 0.79 | 2.97 ± 0.80 | <0.001 |  | 2.87 ± 0.76 | 3.12 ± 0.81 | <0.001 |
| SUA (umol/L) | 409.61 ± 119.08 | 404.59 ± 110.31 | 0.105 |  | 453.31 ± 111.17 | 366.70 ± 99.09 | <0.001 |
| HUA, n(%) | 2088 (53.17%) | 4096 (51.76%) | 0.147 |  | 3146 (58.17%) | 3038 (47.23%) | <0.001 |
| BUN (mmol/L) | 4.99 ± 1.37 | 5.36 ± 1.47 | <0.001 |  | 5.34 ± 1.47 | 5.15 ± 1.42 | <0.001 |
| Serum creatinine (mmol/L) | 62.93 ± 17.22 | 65.10 ± 16.75 | <0.001 |  | 74.93 ± 14.99 | 55.51 ± 12.92 | <0.001 |
| eGFR (ml/min/1.73m^2^) | 101.45 ± 13.77 | 89.12 ± 12.80 | <0.001 |  | 91.43 ± 14.11 | 94.70 ± 14.40 | <0.001 |
| Human serum albumin (g/L) | 47.75 ± 3.91 | 46.36 ± 3.97 | <0.001 |  | 46.54 ± 3.99 | 47.06 ± 4.00 | <0.001 |
| Total bilirubin (mmol/L) | 13.50 (10.40-17.70) | 13.40 (10.30-17.48) | 0.276 |  | 14.30 (11.10-18.90) | 12.70 (9.80-16.40) | <0.001 |
| Direct bilirubin (mmol/L) | 5.10 (4.10-6.40) | 5.20 (4.10-6.60) | <0.001 |  | 5.60 (4.50-7.10) | 4.80 (3.90-6.00) | <0.001 |
| AST (U/L) | 24.00 (20.00-30.00) | 24.00 (20.00-29.00) | 0.105 |  | 25.00 (21.00-31.00) | 24.00 (20.00-29.00) | <0.001 |
| ALT (U/L) | 20.00 (14.00-29.00) | 16.00 (12.00-22.00) | <0.001 |  | 18.00 (13.00-26.00) | 16.00 (12.00-22.00) | <0.001 |
| CHD, n(%) | 95 (2.42%) | 443 (5.60%) | <0.001 |  | 263 (4.86%) | 275 (4.27%) | 0.126 |
| DM, n(%) | 707 (18.00%) | 1357 (17.15%) | 0.247 |  | 833 (15.40%) | 1231 (19.14%) | <0.001 |
| Dyslipidemia, n(%) | 1741 (44.33%) | 2661 (33.62%) | <0.001 |  | 1730 (31.99%) | 2672 (41.54%) | <0.001 |
| Antihypertensive agents, n(%) | 2233 (56.88%) | 5133 (64.87%) | <0.001 |  | 3319 (61.38%) | 4047 (62.92%) | 0.086 |
| Hypoglycemic agents, n(%) | 189 (4.81%) | 365 (4.61%) | 0.626 |  | 206 (3.81%) | 348 (5.41%) | <0.001 |
| Lipid-lowering agents, n(%) | 85 (2.16%) | 200 (2.53%) | 0.225 |  | 122 (2.26%) | 163 (2.53%) | 0.326 |
| Antiplatelet agents, n(%) | 54 (1.38%) | 211 (2.67%) | <0.001 |  | 137 (2.53%) | 128 (1.99%) | 0.046 |
| *Occurrence of follow-up events* |  |  |  |  |  |  |  |
| Incident stroke, n(%) | 16 (0.41%) | 83 (1.05%) | <0.001 |  | 56 (1.04%) | 43 (0.67%) | 0.029 |
| Stroke subtypes, n(%) |  |  | <0.001 |  |  |  | 0.079 |
| Ischemic stroke | 5 (0.13%) | 46 (0.58%) |  |  | 27 (0.50%) | 24 (0.37%) |  |
| Hemorrhagic stroke | 7 (0.18%) | 8 (0.10%) |  |  | 11 (0.20%) | 4 (0.06%) |  |
| Indeterminate stroke | 4 (0.10%) | 29 (0.37%) |  |  | 18 (0.33%) | 15 (0.23%) |  |
| Cause of death, n(%) |  |  | <0.001 |  |  |  | 0.252 |
| Stroke | 4 (0.10%) | 30 (0.38%) |  |  | 19 (0.35%) | 15 (0.23%) |  |
| Heart Disease | 2 (0.05%) | 27 (0.34%) |  |  | 18 (0.33%) | 11 (0.17%) |  |
| Cancer | 4 (0.10%) | 36 (0.45%) |  |  | 21 (0.39%) | 19 (0.30%) |  |
| Respiratory disease | 0 (0.00%) | 4 (0.05%) |  |  | 2 (0.04%) | 2 (0.03%) |  |
| Others | 0 (0.00%) | 17 (0.21%) |  |  | 10 (0.18%) | 7 (0.11%) |  |
| Abbreviations: SBP, systolic blood pressure; DBP, diastolic blood pressure; MAP, mean arterial pressure; HR, heart rate; BMI, body mass index; WHR, waist hip rate; Hcy, homocysteine; FBG, fasting blood glucose; TC, total cholesterol; TG, total triglyceride; HDL-C, high-density lipoprotein cholesterol; LDL-C, low-density lipoprotein cholesterol; SUA, serum uric acid; HUA, hyperuricemia; BUN, blood urea nitrogen; eGFR, estimated glomerular filtration rate; AST, aspartate aminotransferase; ALT, alanine aminotransferase; CHD, coronary heart disease; DM, diabetes mellitus. | | | | | | | |

| \| **Supplement table 3. SUA levels and proportion of HUA betwee non-stroke patients and patients with first stroke** \| \| \| \| \| --- \| --- \| --- \| --- \| \| **Characteristics** \| **First stroke** \| \| \| \| **No** \| **Yes** \| ***P-value*** \| \| Number of subjects (n) \| 3927 \| 7914 \|  \| \| SUA (umol/L) \| 406.16 ± 113.29 \| 417.81 ± 115.22 \| 0.340 \| \| HUA, n(%) \| 6125 (52.16%) \| 59 (59.60%) \| 0.140 \| \| Abbreviations: SUA, serum uric acid; HUA, hyperuricemia. \| \| \| \|   **Supplementary Table 4.** Threshold effect analysis of age and serum uric acid levels using Piece-wise logistic regression. | | | | | | |
| --- | --- | --- | --- | --- | --- | --- | --- | --- | --- | --- | --- | --- | --- | --- | --- | --- | --- | --- | --- | --- | --- | --- | --- | --- | --- | --- | --- | --- | --- | --- | --- | --- | --- |
| Inflection point of age | Number of participants | Event, n(%) | Effect size (*β*) | *95%CI* | P-value | LLR test |
| <57years | 3070 | 411.12 ± 119.25 | -2.97 | (-3.59, -2.35) | <0.001 | <0.001 |
| ≥57years | 8771 | 404.55 ± 111.11 | -1.67 | (-1.99, -1.35) | <0.001 |  |
| Effect: serum uric acid levels; Cause: age.  Adjusted for sex, SBP, DBP, BMI, smoking and drinking status, Hcy, TG, HDL-C, LDL-C, eGFR, AST and ALT. Abbreviations: *CI*, confidence interval; LLR, log-likelihood ratio. | | | | | | |
|  |  |  |  |  |  |  |

| **Supplemenary Table 5. Hazard ratios of serum uric acid level categories for total first stroke events by systolic blood pressure tertiles in different models.** | | | | | | | | | | |
| --- | --- | --- | --- | --- | --- | --- | --- | --- | --- | --- |
| **Variables** | **Event, n(%)** | **Crude Model** | |  | **Model Ⅰ** | |  | **Model Ⅱ** | | ***P-value* for  interaction** |
|  |  | ***HR (95%CI)*** | ***P-value*** |  | ***HR (95%CI)*** | ***P-value*** |  | ***HR (95%CI)*** | ***P-value*** |  |
| *SBP T1 [83.33, 140.67]* |  |  |  |  |  |  |  |  |  |  |
| SUA |  |  |  |  |  |  |  |  |  |  |
| Per *SD* μmol/L increase | 20 (0.51%) | 1.08 (0.71, 1.65) | 0.702 |  | 0.96 (0.61, 1.52) | 0.857 |  | 0.91 (0.53, 1.56) | 0.727^a^ |  |
| HUA |  |  |  |  |  |  |  |  |  |  |
| No | 7 (0.40%) | *Ref* |  |  | *Ref* |  |  | *Ref* |  |  |
| Yes | 13 (0.60%) | 1.48 (0.59, 3.72) | 0.403 |  | 1.40 (0.55, 3.52) | 0.480 |  | 1.50 (0.54, 4.16) | 0.430^b^ |  |
| Quartiles of SUA |  |  |  |  |  |  |  |  |  |  |
| Q1 [93.00, 325.00] | 5 (0.61%) | *Ref* |  |  | *Ref* |  |  | *Ref* |  |  |
| Q2 [326.00, 393.00] | 4 (0.44%) | 0.71 (0.19, 2.65) | 0.610 |  | 0.60 (0.16, 2.28) | 0.450 |  | 0.53 (0.13, 2.10) | 0.368^c^ |  |
| Q3 [394.00, 472.00] | 5 (0.49%) | 0.80 (0.23, 2.77) | 0.722 |  | 0.56 (0.15, 2.06) | 0.387 |  | 0.47 (0.12, 1.85) | 0.282^c^ |  |
| Q4 [473.00, 1029.00] | 6 (0.52%) | 0.84 (0.26, 2.77) | 0.776 |  | 0.55 (0.15, 1.97) | 0.357 |  | 0.39 (0.09, 1.71) | 0.211^c^ |  |
| P for trend |  | 0.873 | |  | 0.439 | |  | 0.264 | |  |
|  |  |  |  |  |  |  |  |  |  |  |
| *SBP T2 [141.00, 154.33]* |  |  |  |  |  |  |  |  |  |  |
| SUA |  |  |  |  |  |  |  |  |  |  |
| Per *SD* μmol/L increase | 29 (0.73%) | 1.02 (0.71, 1.46) | 0.927 |  | 1.01 (0.68, 1.51) | 0.947 |  | 0.85 (0.53, 1.35) | 0.484^d^ |  |
| HUA |  |  |  |  |  |  |  |  |  |  |
| No | 13 (0.67%) | *Ref* |  |  | *Ref* |  |  | *Ref* |  |  |
| Yes | 16 (0.79%) | 1.19 (0.57, 2.48) | 0.644 |  | 1.16 (0.55, 2.45) | 0.695 |  | 0.96 (0.42, 2.18) | 0.914^e^ |  |
| Quartiles of SUA |  |  |  |  |  |  |  |  |  |  |
| Q1 [38.00, 325.80] | 6 (0.58%) | *Ref* |  |  | *Ref* |  |  | *Ref* |  |  |
| Q2 [326.00, 393.30] | 6 (0.61%) | 1.05 (0.34, 3.28) | 0.929 |  | 1.02 (0.32, 3.24) | 0.974 |  | 0.82 (0.25, 2.69) | 0.746^f^ |  |
| Q3 [394.00, 472.00] | 11 (1.12%) | 1.94 (0.72, 5.28) | 0.192 |  | 1.92 (0.67, 5.45) | 0.223 |  | 1.44 (0.48, 4.34) | 0.514^f^ |  |
| Q4 [473.00, 1056.00] | 6 (0.62%) | 1.08 (0.35, 3.37) | 0.891 |  | 1.05 (0.31, 3.48) | 0.943 |  | 0.68 (0.18, 2.60) | 0.574^f^ |  |
| P for trend |  | 0.682 |  |  | 0.76 |  |  | 0.712 |  |  |
|  |  |  |  |  |  |  |  |  |  |  |
| *SBP T3 [154.67, 253.67]* |  |  |  |  |  |  |  |  |  |  |
| SUA |  |  |  |  |  |  |  |  |  |  |
| Per *SD* μmol/L increase | 50 (1.26%) | 1.25 (0.95, 1.63) | 0.107 |  | 1.09 (0.80, 1.49) | 0.569 |  | 1.08 (0.75, 1.55) | 0.678^g^ | 0.673^j^ |
| HUA |  |  |  |  |  |  |  |  |  |  |
| No | 20 (1.01%) | *Ref* |  |  | *Ref* |  |  | *Ref* |  | 0.862^k^ |
| Yes | 30 (1.51%) | 1.50 (0.85, 2.65) | 0.164 |  | 1.34 (0.75, 2.38) | 0.324 |  | 1.38 (0.73, 2.59) | 0.322^h^ |  |
| Quartiles of SUA |  |  |  |  |  |  |  |  |  |  |
| Q1 [127.00, 325.00] | 11 (0.99%) | *Ref* |  |  | *Ref* |  |  | *Ref* |  | 0.821^l^ |
| Q2 [326.00, 393.00] | 13 (1.26%) | 1.27 (0.57, 2.85) | 0.562 |  | 1.03 (0.45, 2.35) | 0.939 |  | 0.96 (0.41, 2.25) | 0.933^i^ |  |
| Q3 [394.00, 472.00] | 13 (1.35%) | 1.36 (0.61, 3.06) | 0.452 |  | 0.96 (0.41, 2.24) | 0.918 |  | 0.84 (0.34, 2.07) | 0.705^i^ |  |
| Q4 [473.00, 907.00] | 13 (1.52%) | 1.53 (0.68, 3.44) | 0.300 |  | 0.99 (0.41, 2.37) | 0.977 |  | 0.86 (0.32, 2.31) | 0.760^i^ |  |
| P for trend |  | 0.301 | |  | 0.942 | |  | 0.730 | |  |
| Abbreviations: SBP, systolic blood pressure; SUA, serum uric acid; HUA, hyperuricemia; *Ref*, reference; *HR*, hazard ratio; *CI*, confidence interval; *SD*, standard deviation. Model Ⅰ adjusted for age, sex, DBP and HR.  Model Ⅱ: ^a^adjusted for age, sex, DBP, HR, BMI, WHR, smoking and drinking status, Hcy, TG, HDL-C, LDL-C, eGFR, AST, ALT and antiplatelet agents. ^b^adjusted for sex, HR, BMI, WHR, drinking status, LDL-C, eGFR, AST, ALT and antiplatelet agents. ^c^adjusted for sex, DBP, HR, BMI, WHR, smoking and drinking status, Hcy, TG, HDL-C, LDL-C, eGFR, AST, ALT and antiplatelet agents. ^d^adjusted for age, sex, DBP, HR, BMI, WHR, smoking and drinking status, Hcy, TG, HDL-C, LDL-C, eGFR, total bilirubin, ALT, DM, antihypertensive agents, lipid-lowering agents and antiplatelet agents. ^e^adjusted for age, sex, DBP, HR, BMI, WHR, smoking and drinking status, Hcy, TG, HDL-C, LDL-C, eGFR, ALT, DM, antihypertensive agents, lipid-lowering agents and antiplatelet agents. ^f^adjusted for age, sex, DBP, HR, BMI, WHR, smoking and drinking status, Hcy, TG, HDL-C, LDL-C, eGFR, total bilirubin, ALT, DM, antihypertensive agents and lipid-lowering agents. ^g^adjusted for age, sex, DBP, BMI, WHR, smoking and drinking status, Hcy, TG, HDL-C, eGFR and AST. ^h^adjusted for age, sex, DBP, BMI, WHR, smoking and drinking status, Hcy, TG, HDL-C, LDL-C and eGFR. ^i^adjusted for age, sex, DBP, BMI, WHR, smoking and drinking status, Hcy, TG, HDL-C, LDL-C, eGFR, AST, ALT, antihypertensive agents and lipid-lowering agents. ^j^adjusted for age, sex, DBP, HR, BMI, WHR, smoking and drinking status, Hcy, TG, HDL-C, LDL-C, eGFR, total bilirubin, ALT, DM, antiplatelet agents and the interaction terms for following variables: age, sex, DBP, BMI, WHR, smoking and drinking status, Hcy, TG, HDL-C, LDL-C, eGFR, total bilirubin and ALT. ^k^adjusted for age, sex, DBP, HR, BMI, smoking status, Hcy, TG, eGFR, ALT, antiplatelet agents and the interaction terms for following variables: age, sex, DBP, HR, BMI, smoking status, Hcy, TG, eGFR, ALT, antiplatelet agents. ^l^adjusted for age, sex, DBP, HR, BMI, WHR, smoking and drinking status, Hcy, TG, HDL-C, LDL-C, eGFR, total bilirubin, ALT, AST, antiplatelet agents and the interaction terms for following variables: age, sex, DBP, HR, BMI, WHR, smoking and drinking status, Hcy, TG, HDL-C, LDL-C, eGFR, total bilirubin, ALT, AST, antiplatelet agents. | | | | | | | | | | |

| **Supplemenary Table 6. Hazard ratios of serum uric acid level categories for total first stroke events by diastolic blood pressure tertiles in different models.** | | | | | | | | | | |
| --- | --- | --- | --- | --- | --- | --- | --- | --- | --- | --- |
| **Variables** | **Event, n(%)** | **Crude Model** | |  | **Model Ⅰ** | |  | **Model Ⅱ** | | ***P-value* for  interaction** |
|  |  | ***HR (95%CI)*** | ***P-value*** |  | ***HR (95%CI)*** | ***P-value*** |  | ***HR (95%CI)*** | ***P-value*** |  |
| *DBP T1 [41.67, 84.67]* |  |  |  |  |  |  |  |  |  |  |
| SUA |  |  |  |  |  |  |  |  |  |  |
| Per *SD* μmol/L increase | 26 (0.68%) | 1.45 (1.02, 2.06) | 0.038 |  | 1.42 (0.97, 2.08) | 0.074 |  | 1.24 (0.82, 1.89) | 0.303^a^ |  |
| HUA |  |  |  |  |  |  |  |  |  |  |
| No | 9 (0.48%) | *Ref* |  |  | *Ref* |  |  | *Ref* |  |  |
| Yes | 17 (0.88%) | 1.84 (0.82, 4.15) | 0.139 |  | 1.73 (0.76, 3.91) | 0.189 |  | 1.49 (0.62, 3.60) | 0.371^a^ |  |
| Quartiles of SUA |  |  |  |  |  |  |  |  |  |  |
| Q1 [118.00, 325.00] | 4 (0.40%) | *Ref* |  |  | *Ref* |  |  | *Ref* |  |  |
| Q2 [326.00, 393.00] | 6 (0.60%) | 1.52 (0.43, 5.41) | 0.516 |  | 1.42 (0.40, 5.12) | 0.588 |  | 1.23 (0.33, 4.62) | 0.760^b^ |  |
| Q3 [394.00, 472.00] | 5 (0.55%) | 1.38 (0.37, 5.17) | 0.629 |  | 1.24 (0.32, 4.75) | 0.758 |  | 1.02 (0.25, 4.21) | 0.977^b^ |  |
| Q4 [473.00, 965.00] | 11 (1.24%) | 3.15 (1.00, 9.94) | 0.050 |  | 2.78 (0.84, 9.27) | 0.095 |  | 2.18 (0.56, 8.48) | 0.262^b^ |  |
| P for trend |  | 0.035 | |  | 0.071 | |  | 0.214 | |  |
|  |  |  |  |  |  |  |  |  |  |  |
| *DBP T2 [85.00, 93.33]* |  |  |  |  |  |  |  |  |  |  |
| SUA |  |  |  |  |  |  |  |  |  |  |
| Per *SD* μmol/L increase | 33 (0.81%) | 1.08 (0.78, 1.52) | 0.634 |  | 1.01 (0.69, 1.47) | 0.965 |  | 1.05 (0.68, 1.65) | 0.816^c^ |  |
| HUA |  |  |  |  |  |  |  |  |  |  |
| No | 13 (0.66%) | *Ref* |  |  | *Ref* |  |  | *Ref* |  |  |
| Yes | 20 (0.94%) | 1.43 (0.71, 2.89) | 0.316 |  | 1.41 (0.69, 2.85) | 0.344 |  | 1.64 (0.77, 3.51) | 0.202^d^ |  |
| Quartiles of SUA |  |  |  |  |  |  |  |  |  |  |
| Q1 [38.00, 325.00] | 10 (0.99%) | *Ref* |  |  | *Ref* |  |  | *Ref* |  |  |
| Q2 [326.00, 393.30] | 5 (0.47%) | 0.47 (0.16, 1.39) | 0.174 |  | 0.39 (0.13, 1.16) | 0.090 |  | 0.38 (0.12, 1.16) | 0.089^e^ |  |
| Q3 [394.00, 472.00] | 11 (1.07%) | 1.08 (0.46, 2.56) | 0.857 |  | 0.80 (0.32, 2.00) | 0.634 |  | 0.76 (0.29, 2.00) | 0.574^e^ |  |
| Q4 [473.00, 1029.00] | 7 (0.71%) | 0.71 (0.27, 1.88) | 0.491 |  | 0.51 (0.18, 1.47) | 0.214 |  | 0.51 (0.15, 1.67) | 0.262^e^ |  |
| P for trend |  | 0.805 |  |  | 0.419 |  |  | 0.456 |  |  |
|  |  |  |  |  |  |  |  |  |  |  |
| *DBP T3 [93.67, 142.33]* |  |  |  |  |  |  |  |  |  |  |
| SUA |  |  |  |  |  |  |  |  |  |  |
| Per *SD* μmol/L increase | 40 (1.01%) | 0.91 (0.67, 1.25) | 0.557 |  | 0.85 (0.59, 1.22) | 0.375 |  | 0.76 (0.50, 1.16) | 0.198^f^ | 0.313^i^ |
| HUA |  |  |  |  |  |  |  |  |  |  |
| No | 18 (0.99%) | *Ref* |  |  | *Ref* |  |  | *Ref* |  | 0.519^j^ |
| Yes | 22 (1.03%) | 1.04 (0.56, 1.94) | 0.905 |  | 1.03 (0.55, 1.95) | 0.924 |  | 0.98 (0.49, 1.98) | 0.961^g^ |  |
| Quartiles of SUA |  |  |  |  |  |  |  |  |  |  |
| Q1 [108.00, 325.80] | 8 (0.85%) | *Ref* |  |  | *Ref* |  |  | *Ref* |  | 0.152^k^ |
| Q2 [326.00, 393.00] | 12 (1.37%) | 1.61 (0.66, 3.97) | 0.297 |  | 1.42 (0.57, 3.57) | 0.455 |  | 1.31 (0.51, 3.37) | 0.574^h^ |  |
| Q3 [394.00, 472.00] | 13 (1.27%) | 1.50 (0.62, 3.62) | 0.373 |  | 1.22 (0.47, 3.13) | 0.683 |  | 0.99 (0.36, 2.68) | 0.982^h^ |  |
| Q4 [473.00, 1056.00] | 7 (0.64%) | 0.75 (0.27, 2.07) | 0.574 |  | 0.64 (0.21, 1.91) | 0.422 |  | 0.48 (0.14, 1.60) | 0.233^h^ |  |
| P for trend |  | 0.462 | |  | 0.295 | |  | 0.144 | |  |
| Abbreviations: DBP, diastolic blood pressure; SUA, serum uric acid; HUA, hyperuricemia; *Ref*, reference; *HR*, hazard ratio; *CI*, confidence interval; *SD*, standard deviation. Model Ⅰ adjusted for age, sex, SBP and HR.  Model Ⅱ: ^a^adjusted for age, SBP, HR, BMI, drinking status, Hcy, TG and eGFR. ^b^adjusted for age, sex, SBP, HR, BMI, WHR, smoking and drinking status, Hcy, TG, HDL-C, LDL-C, eGFR, total bilirubin, ALT, DM, antihypertensive agents, lipid-lowering agents and antiplatelet agents. ^c^adjusted for age, sex, SBP, HR, BMI, smoking and drinking status, Hcy, TG, HDL-C, LDL-C, eGFR, AST, ALT and DM. ^d^djusted for age, sex, HR, BMI, smoking status, TG and eGFR. ^e^adjusted for age, sex, SBP, HR, BMI, smoking and drinking status, Hcy, TG, HDL-C, LDL-C, eGFR, AST, ALT, DM, DM and antihypertensive agents. ^f^adjusted for age, sex, SBP, BMI, WHR, smoking status, Hcy, TG, HDL-C, LDL-C, eGFR, AST and ALT. ^g^adjusted for age, sex, SBP, BMI, WHR, smoking and drinking status, Hcy, TG, HDL-C, LDL-C, eGFR, total bilirubin, AST, ALT, antihypertensive agents and antiplatelet agents. ^h^adjusted for age, sex, SBP, HR, BMI, WHR, smoking and drinking status, Hcy, TG, HDL-C, LDL-C, eGFR, total bilirubin, AST, ALT, DM, antihypertensive agents, lipid-lowering agents and antiplatelet agents. ^i^adjusted for age, sex, SBP, HR, BMI, WHR, smoking and drinking status, Hcy, TG, HDL-C, LDL-C, eGFR, total bilirubin, ALT, DM, antiplatelet agents and the interaction terms for following variables: sex, SBP, HR, BMI, WHR, smoking and drinking status, Hcy, TG, LDL-C, eGFR, total bilirubin, ALT, DM. ^j^adjusted for age, sex, SBP, HR, BMI, smoking status, Hcy, TG, eGFR, ALT, antiplatelet agents and the interaction terms for following variables: age, sex, SBP, HR, BMI, smoking status, Hcy, TG, eGFR, ALT. ^k^adjusted for age, sex, SBP, HR, BMI, WHR, smoking and drinking status, Hcy, TG, HDL-C, LDL-C, eGFR, total bilirubin, ALT, AST, antiplatelet agents and the interaction terms for following variables: age, sex, SBP, HR, BMI, WHR, smoking and drinking status, Hcy, TG, HDL-C, LDL-C, eGFR, total bilirubin, ALT, AST, antiplatelet agents. | | | | | | | | | | |

| **Supplemenary Table 7. Hazard ratios of serum uric acid level categories for total first stroke events by body mass index tertiles in different models.** | | | | | | | | | | |
| --- | --- | --- | --- | --- | --- | --- | --- | --- | --- | --- |
| **Variables** | **Event, n(%)** | **Crude Model** | |  | **Model Ⅰ** | |  | **Model Ⅱ** | | ***P-value* for  interaction** |
|  |  | ***HR (95%CI)*** | ***P-value*** |  | ***HR (95%CI)*** | ***P-value*** |  | ***HR (95%CI)*** | ***P-value*** |  |
| *BMI T1 [12.45, 22.09]* |  |  |  |  |  |  |  |  |  |  |
| SUA |  |  |  |  |  |  |  |  |  |  |
| Per *SD* μmol/L increase | 46 (1.17%) | 1.15 (0.86, 1.54) | 0.351 |  | 1.06 (0.75, 1.49) | 0.728 |  | 1.05 (0.71, 1.54) | 0.817^a^ |  |
| HUA |  |  |  |  |  |  |  |  |  |  |
| No | 22 (0.98%) | *Ref* |  |  | *Ref* |  |  | *Ref* |  |  |
| Yes | 24 (1.42%) | 1.46 (0.81, 2.61) | 0.205 |  | 1.32 (0.73, 2.39) | 0.354 |  | 1.34 (0.71, 2.50) | 0.364^b^ |  |
| Quartiles of SUA |  |  |  |  |  |  |  |  |  |  |
| Q1 [38.00, 325.00] | 12 (0.98%) | *Ref* |  |  | *Ref* |  |  | *Ref* |  |  |
| Q2 [326.00, 393.00] | 11 (1.03%) | 1.05 (0.46, 2.40) | 0.903 |  | 0.92 (0.40, 2.15) | 0.852 |  | 0.88 (0.37, 2.10) | 0.780^c^ |  |
| Q3 [394.00, 472.00] | 14 (1.52%) | 1.56 (0.72, 3.38) | 0.264 |  | 1.20 (0.52, 2.77) | 0.674 |  | 1.14 (0.47, 2.75) | 0.770^c^ |  |
| Q4 [473.00, 915.00] | 9 (1.21%) | 1.24 (0.52, 2.95) | 0.631 |  | 0.96 (0.38, 2.46) | 0.938 |  | 0.91 (0.32, 2.55) | 0.851^c^ |  |
| P for trend |  | 0.451 | |  | 0.94 | |  | 0.966 | |  |
|  |  |  |  |  |  |  |  |  |  |  |
| *BMI T2 [22.09, 25.07]* |  |  |  |  |  |  |  |  |  |  |
| SUA |  |  |  |  |  |  |  |  |  |  |
| Per *SD* μmol/L increase | 26 (0.66%) | 1.10 (0.75, 1.62) | 0.612 |  | 1.12 (0.73, 1.70) | 0.607 |  | 0.96 (0.58, 1.58) | 0.872^d^ |  |
| HUA |  |  |  |  |  |  |  |  |  |  |
| No | 10 (0.52%) | *Ref* |  |  | *Ref* |  |  | *Ref* |  |  |
| Yes | 16 (0.79%) | 1.50 (0.68, 3.32) | 0.314 |  | 1.49 (0.67, 3.33) | 0.326 |  | 1.21 (0.51, 2.89) | 0.664^e^ |  |
| Quartiles of SUA |  |  |  |  |  |  |  |  |  |  |
| Q1 [108.00, 325.80] | 6 (0.61%) | *Ref* |  |  | *Ref* |  |  | *Ref* |  |  |
| Q2 [326.00, 393.30] | 6 (0.59%) | *0.98 (0.31, 3.04)* | 0.968 |  | 0.97 (0.31, 3.05) | 0.953 |  | 0.84 (0.25, 2.77) | 0.771^f^ |  |
| Q3 [394.00, 472.00] | 7 (0.71%) | 1.18 (0.39, 3.52) | 0.770 |  | 1.15 (0.37, 3.62) | 0.808 |  | 0.83 (0.25, 2.81) | 0.770^f^ |  |
| Q4 [473.00, 1056.00] | 7 (0.72%) | 1.19 (0.40, 3.56) | 0.753 |  | 1.21 (0.37, 3.97) | 0.758 |  | 0.84 (0.22, 3.22) | 0.799^f^ |  |
| P for trend |  | 0.692 |  |  | 0.705 |  |  | 0.832 |  |  |
|  |  |  |  |  |  |  |  |  |  |  |
| *BMI T3 [25.07, 62.88]* |  |  |  |  |  |  |  |  |  |  |
| SUA |  |  |  |  |  |  |  |  |  |  |
| Per *SD* μmol/L increase | 27 (0.68%) | 1.18 (0.84, 1.67) | 0.341 |  | 1.04 (0.71, 1.54) | 0.833 |  | 0.95 (0.61, 1.48) | 0.809^g^ | 0.995^i^ |
| HUA |  |  |  |  |  |  |  |  |  |  |
| No | 8 (0.54%) | *Ref* |  |  | *Ref* |  |  | *Ref* |  | 0.987^j^ |
| Yes | 19 (0.77%) | 1.45 (0.63, 3.32) | 0.378 |  | 1.32 (0.57, 3.05) | 0.521 |  | 1.15 (0.47, 2.84) | 0.757^h^ |  |
| Quartiles of SUA |  |  |  |  |  |  |  |  |  |  |
| Q1 [93.00, 325.00] | 4 (0.53%) | *Ref* |  |  | *Ref* |  |  | *Ref* |  | 0.992^k^ |
| Q2 [326.00, 393.00] | 6 (0.70%) | 1.31 (0.37, 4.66) | 0.676 |  | 1.07 (0.30, 3.84) | 0.923 |  | 1.02 (0.27, 3.83) | 0.977^f^ |  |
| Q3 [394.00, 472.00] | 8 (0.75%) | 1.41 (0.42, 4.70) | 0.575 |  | 1.01 (0.29, 3.50) | 0.992 |  | 0.85 (0.23, 3.15) | 0.808^f^ |  |
| Q4 [473.00, 965.00] | 9 (0.71%) | 1.33 (0.41, 4.35) | 0.632 |  | 0.85 (0.24, 3.01) | 0.801 |  | 0.63 (0.16, 2.54) | 0.516^f^ |  |
| P for trend |  | 0.691 | |  | 0.710 | |  | 0.409 | |  |
| Abbreviations: BMI, body mass index; SUA, serum uric acid; HUA, hyperuricemia; *Ref*, reference; *HR*, hazard ratio; *CI*, confidence interval; *SD*, standard deviation. Model Ⅰ adjusted for age, sex, SBP, DBP and HR. Model Ⅱ: ^a^adjusted for age, sex, SBP, DBP, HR, BMI, WHR, smoking and drinking status, Hcy, HDL-C, eGFR, AST, ALT, antihypertensive agents and antiplatelet agents. ^b^adjusted for age, sex, SBP, DBP, HR, Hcy, HDL-C and eGFR. ^c^adjusted for age, sex, SBP, DBP, HR, WHR, smoking and drinking status, Hcy, LDL-C, eGFR, total bilirubin, AST, ALT, DM, antihypertensive agents and antiplatelet agents. ^d^adjusted for age, sex, SBP, DBP, HR, WHR, smoking and drinking status, Hcy, TG, HDL-C, LDL-C, eGFR, total bilirubin, AST, ALT, DM, antihypertensive agents and antiplatelet agents. ^e^adjusted for age, SBP, DBP, HR, WHR, smoking status, Hcy, TG, eGFR, AST, ALT, DM, antihypertensive agents, lipid-lowering agents and antiplatelet agents. ^f^adjusted for age, sex, SBP, DBP, HR, WHR, smoking and drinking status, Hcy, TG, HDL-C, LDL-C, eGFR, total bilirubin, AST, ALT, DM, antihypertensive agents, lipid-lowering agents and antiplatelet agents. ^g^adjusted for age, sex, SBP, DBP, WHR, smoking and drinking status, Hcy, TG, LDL-C, eGFR, total bilirubin, AST, ALT, antihypertensive agents, lipid-lowering agents and antiplatelet agents. ^h^adjusted for age, sex, SBP, DBP, WHR, smoking status, TG, LDL-C, eGFR, total bilirubin, AST, ALT and antiplatelet agents. ^i^adjusted for age, sex, SBP, DBP, HR, WHR, smoking and drinking status, Hcy, TG, HDL-C, LDL-C, eGFR, total bilirubin, ALT, DM, antiplatelet agents and the interaction terms for following variables: sex, HR, WHR, smoking and drinking status, Hcy, TG, HDL-C, LDL-C, eGFR, total bilirubin, ALT, antiplatelet agents. ^j^adjusted for age, sex, SBP, DBP, HR, smoking status, Hcy, TG, eGFR, ALT, antiplatelet agents and the interaction terms for following variables: age, sex, SBP, DBP, HR, smoking status, Hcy, TG, eGFR, ALT. ^k^adjusted for age, sex, SBP, HR, WHR, smoking and drinking status, Hcy, TG, HDL-C, LDL-C, eGFR, total bilirubin, ALT, AST, antiplatelet agents and the interaction terms for following variables: age, sex, SBP, HR, WHR, smoking and drinking status, Hcy, TG, HDL-C, LDL-C, eGFR, total bilirubin, ALT, AST, antiplatelet agents. | | | | | | | | | | |

| **Supplemenary Table 8. Hazard ratios of serum uric acid level categories for total first stroke events by central obesity in different models.** | | | | | | | | | | |
| --- | --- | --- | --- | --- | --- | --- | --- | --- | --- | --- |
| **Variables** | **Event, n(%)** | **Crude Model** | |  | **Model Ⅰ** | |  | **Model Ⅱ** | | ***P-value* for  interaction** |
|  |  | ***HR (95%CI)*** | ***P-value*** |  | ***HR (95%CI)*** | ***P-value*** |  | ***HR (95%CI)*** | ***P-value*** |  |
| *Non-central obesity* |  |  |  |  |  |  |  |  |  |  |
| SUA |  |  |  |  |  |  |  |  |  |  |
| Per *SD* μmol/L increase | 37 (1.08%) | 1.16 (0.83, 1.61) | 0.392 |  | 1.13 (0.77, 1.66) | 0.526 |  | 0.97 (0.62, 1.54) | 0.906^a^ |  |
| HUA |  |  |  |  |  |  |  |  |  |  |
| No | 15 (0.80%) | *Ref* |  |  | *Ref* |  |  | *Ref* |  |  |
| Yes | 22 (1.42%) | 1.79 (0.93, 3.46) | 0.084 |  | 1.70 (0.87, 3.31) | 0.121 |  | 1.44 (0.71, 2.93) | 0.315^b^ |  |
| Quartiles of SUA |  |  |  |  |  |  |  |  |  |  |
| Q1 [127.00, 325.00] | 7 (0.78%) | *Ref* |  |  | *Ref* |  |  | *Ref* |  |  |
| Q2 [326.00, 393.30] | 10 (1.12%) | 1.44 (0.55, 3.80) | 0.462 |  | 1.21 (0.45, 3.29) | 0.708 |  | 0.98 (0.35, 2.73) | 0.964^c^ |  |
| Q3 [394.00, 472.00] | 12 (1.36%) | 1.74 (0.68, 4.45) | 0.245 |  | 1.54 (0.56, 4.20) | 0.399 |  | 1.16 (0.40, 3.36) | 0.784^c^ |  |
| Q4 [473.00, 987.00] | 8 (1.07%) | 1.37 (0.50, 3.80) | 0.544 |  | 1.18 (0.40, 3.54) | 0.761 |  | 0.78 (0.23, 2.67) | 0.690^c^ |  |
| P for trend |  | 0.519 | |  | 0.744 | |  | 0.696 | |  |
|  |  |  |  |  |  |  |  |  |  |  |
| *Central obesity* |  |  |  |  |  |  |  |  |  |  |
| SUA |  |  |  |  |  |  |  |  |  |  |
| Per SD μmol/L increase | 62 (0.74%) | 1.10 (0.87, 1.39) | 0.446 |  | 1.04 (0.79, 1.35) | 0.790 |  | 0.98 (0.72, 1.33) | 0.890^d^ | 0.940^g^ |
| HUA |  |  |  |  |  |  |  |  |  |  |
| No | 25 (0.66%) | *Ref* |  |  | *Ref* |  |  | *Ref* |  |  |
| Yes | 37 (0.80%) | 1.21 (0.73, 2.01) | 0.466 |  | 1.16 (0.69, 1.95) | 0.570 |  | 1.09 (0.62, 1.91) | 0.760^e^ | 0.622^h^ |
| Quartiles of SUA |  |  |  |  |  |  |  |  |  |  |
| Q1 [38.00, 325.80] | 15 (0.73%) | *Ref* |  |  | *Ref* |  |  | *Ref* |  | 0.930^i^ |
| Q2 [326.00, 393.00] | 13 (0.64%) | 0.87 (0.42, 1.84) | 0.724 |  | 0.82 (0.38, 1.73) | 0.597 |  | 0.78 (0.36, 1.68) | 0.526^f^ |  |
| Q3 [394.00, 472.00] | 17 (0.82%) | 1.13 (0.56, 2.26) | 0.74 |  | 0.94 (0.45, 1.94) | 0.859 |  | 0.80 (0.37, 1.73) | 0.572^f^ |  |
| Q4 [473.00, 1056.00] | 17 (0.76%) | 1.05 (0.52, 2.11) | 0.891 |  | 0.87 (0.40, 1.85) | 0.712 |  | 0.71 (0.30, 1.65) | 0.420^f^ |  |
| P for trend |  | 0.751 | |  | 0.808 | |  | 0.471 | |  |
| Abbreviations: SUA, serum uric acid; HUA, hyperuricemia; *Ref*, reference; *HR*, hazard ratio; *CI*, confidence interval; *SD*, standard deviation. Model Ⅰ adjusted for age, sex, SBP, DBP and HR. Model Ⅱ: ^a^adjusted for age, sex, SBP, DBP, HR, BMI, drinking status, Hcy, TG, HDL-C, eGFR, total bilirubin, AST, ALT, DM, lipid-lowering agents and antiplatelet agents. ^b^adjusted for age, SBP, BMI, Hcy and eGFR. ^c^adjusted for age, sex, SBP, HR, BMI, smoking and drinking status, Hcy, TG, HDL-C, LDL-C, eGFR, total bilirubin, AST, ALT, antihypertensive agents and antiplatelet agents. ^d^adjusted for age, sex, SBP, DBP, HR, BMI, smoking and drinking status, Hcy, TG, HDL-C, LDL-C, eGFR, AST, ALT, lipid-lowering agents and antiplatelet agents. ^e^adjusted for age, sex, SBP, DBP, HR, BMI, smoking and drinking status, Hcy, TG, HDL-C, LDL-C, eGFR, AST, ALT and antiplatelet agents. ^f^adjusted for age, sex, SBP, DBP, HR, BMI, smoking and drinking status, Hcy, TG, HDL-C, LDL-C, eGFR, total bilirubin, AST, ALT, DM, lipid-lowering agents and antiplatelet agents. ^g^adjusted for age, sex, SBP, DBP, HR, BMI, smoking and drinking status, Hcy, TG, HDL-C, LDL-C, eGFR, total bilirubin, ALT, DM, antiplatelet agents and the interaction terms for following variables: age, sex, SBP, DBP, HR, BMI, smoking and drinking status, Hcy, TG, HDL-C, LDL-C, eGFR, total bilirubin, ALT, antiplatelet agents. ^h^adjusted for age, sex, SBP, DBP, HR, BMI, smoking status, Hcy, TG, eGFR, ALT, antiplatelet agents and the interaction terms for following variables: age, sex, SBP, DBP, HR, BMI, smoking status, Hcy, eGFR, ALT, antiplatelet agents. ^i^adjusted for age, sex, SBP, HR, BMI, smoking and drinking status, Hcy, TG, HDL-C, LDL-C, eGFR, total bilirubin, ALT, AST, antiplatelet agents and the interaction terms for following variables: age, sex, SBP, HR, BMI, smoking and drinking status, Hcy, TG, HDL-C, LDL-C, eGFR, total bilirubin, ALT, AST, antiplatelet agents. | | | | | | | | | | |
